# Obesity Induces DNA Damage in Mammary Epithelial Cells Exacerbated by Acrylamide Treatment through CYP2E1-Mediated Oxidative Stress

**DOI:** 10.3390/toxics12070484

**Published:** 2024-07-02

**Authors:** Brenna Walton, Noah Kaplan, Brooke Hrdlicka, Kavi Mehta, Lisa M. Arendt

**Affiliations:** 1 Molecular and Environmental Toxicology, University of Wisconsin-Madison, Madison, WI 53715, USA; 2 Comparative Biosciences, University of Wisconsin-Madison, Madison, WI 53715, USA

**Keywords:** obesity, acrylamide, glycidamide, breast cancer, mammary epithelial cells, DNA damage, oxidative stress, CYP2E1, obesogen, Western diet

## Abstract

Obesity and environmental toxins are risk factors for breast cancer; however, there is limited knowledge on how these risk factors interact to promote breast cancer. Acrylamide, a probable carcinogen and obesogen, is a by-product in foods prevalent in the obesity-inducing Western diet. Acrylamide is metabolized by cytochrome P450 2E1 (CYP2E1) to the genotoxic epoxide, glycidamide, and is associated with an increased risk for breast cancer. To investigate how acrylamide and obesity interact to increase breast cancer risk, female mice were fed a low-fat (LFD) or high-fat diet (HFD) and control water or water supplemented with acrylamide at levels similar to the average daily exposure in humans. While HFD significantly enhanced weight gain in mice, the addition of acrylamide did not significantly alter body weights compared to respective controls. Mammary epithelial cells from obese, acrylamide-treated mice had increased DNA strand breaks and oxidative DNA damage compared to all other groups. In vitro, glycidamide-treated COMMA-D cells showed significantly increased DNA strand breaks, while acrylamide-treated cells demonstrated significantly higher levels of intracellular reactive oxygen species. The knockdown of CYP2E1 rescued the acrylamide-induced oxidative stress. These studies suggest that long-term acrylamide exposure through foods common in the Western diet may enhance DNA damage and the CYP2E1-induced generation of oxidative stress in mammary epithelial cells, potentially enhancing obesity-induced breast cancer risk.

## 1. Introduction

Breast cancer is the second most diagnosed cancer in women [1]. In the United States, approximately one in eight women will receive a breast cancer diagnosis in their lifetime [2]. Family history of breast cancer, sex, age at first menarche, and age at menopause are all strong risk factors for breast cancer [3,4]. Additionally, there are modifiable lifestyle factors that contribute to breast cancer risk, including lack of physical activity, obesity, and chemical exposures such as alcohol, hormone replacement therapy, and carcinogens [3,4]. Individual risk factors may interact with each other to further amplify breast cancer risk. For instance, obesity increases breast cancer risk in individuals with other risk factors, such as familial history of breast cancer [5] or ethnicity [6]. Rates of obesity have tripled since 1970 [7], and chemical production has increased fifty-fold since 1950 [8], indicating a rise in modifiable risk factor exposure and highlighting a need to understand how these risk factors impact the breast to promote tumorigenesis.

Women with obesity are at risk for different subtypes of breast cancer depending on whether they have reached menopause. Premenopausal women with obesity are at an increased risk for triple-negative breast cancer, which lacks the expression of estrogen receptor (ER), progesterone receptor, and human epidermal growth factor receptor 2 [9,10,11], while postmenopausal women with obesity have an increased risk for ER^+^ breast cancer [12,13]. Women with obesity tend to develop more aggressive breast cancers regardless of menopausal status [9,14,15,16]. Within breast tissue, obesity alters mammary epithelial cells to increase ER^+^ luminal cells, decrease basal/myoepithelial cells, and enhance stem and progenitor activity [17]. This indicates obesity may predispose individuals to breast cancer by expanding the epithelial populations that give rise to the most common subtypes of breast cancer [18,19,20,21]. Additionally, obesity increases levels of adipokines and cytokines secreted by adipocytes, leading to enhanced macrophage recruitment and chronic inflammation in the mammary gland [17,22,23,24,25]. These studies suggest that obesity changes the mammary microenvironment to create a supportive niche for tumorigenesis. Further, the changes to the mammary gland as a result of obesity may increase the susceptibility to chemically induced carcinogenesis; however, more research is needed to investigate this hypothesis.

Acrylamide is classified as a probable carcinogen [26] and is produced as a by-product of cooking starchy foods at high temperatures [27]. The obesity-inducing Western diet is made up of foods that have high acrylamide contents [28,29], suggesting that individuals with obesity may be exposed to higher levels of acrylamide. Current estimates suggest that chronic dietary exposure of adolescents and adults is, on average, between 0.4 and 0.9 µg/kg body weight per day [30]. Recommendations to reduce acrylamide production in food are provided by the United States Federal Drug Administration [31]. The European Food Safety Authority concluded that margins of exposure indicate a concern for neoplastic effects based on animal evidence [30], and European companies selling foods containing acrylamide are required to report acrylamide levels due to its adverse health effects [32]. Recently, acrylamide exposure was found to enhance weight gain in mice [33] and zebrafish [34], as well as increase fat droplet accumulation in differentiated 3T3-L1 cultured adipocytes [33]. These studies suggest that acrylamide acts as an obesogen to enhance obesity in these models. Acrylamide is metabolized by the cytochrome P450 enzyme 2E1 (CYP2E1) to glycidamide or conjugated to glutathione for excretion [35,36]. Glycidamide is a genotoxic epoxide that forms DNA adducts at the N7 position of guanine and N3 position of adenine [37,38,39] and can increase tumor formation with long-term exposure in mice and rats [40]. Acrylamide exposure also leads to elevated levels of oxidative stress in a variety of tissues in vivo [41,42,43,44,45,46], although the mechanism for generation of oxidative stress has not been identified. Oxidative stress leads to the generation of reactive oxygen species (ROS) that can react with RNA and DNA to form adducts. Epidemiological studies suggest acrylamide exposure increases breast cancer risk in women [47,48,49,50], potentially through glycidamide-induced DNA damage. However, interactions of acrylamide with other breast cancer risk factors have not been explored.

Significant questions remain regarding the role of acrylamide in promoting obesity and how exposure to acrylamide contributes to breast cancer risk. Here, a high-fat (HFD) model of obesity and chronic exposure to acrylamide in mice was utilized. The dose of acrylamide administered was similar to acrylamide exposure levels observed in humans [30]. Given the role of acylamide as a potential obesogen, the potential for acrylamide to enhance weight gain was examined in mice fed either a low-fat diet (LFD) or HFD. To identify potential mechanisms of how obesity and the Western diet may contribute to increased breast cancer risk, interactions between acrylamide and obesity to induce DNA damage were investigated in the mammary gland and in vitro, utilizing a model of mammary epithelial cells.

## 2. Materials and Methods

### 2.1. Animal Studies

All animal procedures were approved by the University of Wisconsin Institutional Animal Care and Use Committee, per guidelines published by the National Institute of Health (NIH) Guide for the Care and Use of Laboratory Animals (Protocol No. V005188). Female Friend Virus B NIH (FVB/N) mice were purchased from Taconic Biosciences (Germantown, MA, USA) and housed in facilities accredited by the Association for Assessment and Accreditation of Laboratory Animal Care International. Three-week-old FVB/N female mice were fed a LFD (16% kcal from fat, Teklad Global, 2020X, Waldschmidt & Sons, Madison, WI, USA) or HFD (60% kcal from fat, Test Diet #58Y1, Waldschmidt & Sons, Madison, USA) and were provided with sterile filtered water or water supplemented with 0.7 mM acrylamide (A9099, Sigma-Aldrich, St. Louis, MI, USA) for 16 weeks. This concentration of acrylamide in the water provides a chronic exposure of 0.64–0.66 μg/kg body weight per day, similar to that observed in humans [30]. Food and water were provided ad libitum. Body weights were measured weekly. After 16 weeks, mice were injected with 200 mg/kg body weight of 5′-bromo-2′-deoxyuridine (BrdU; AC228590010, Fisher Scientific, Hampton, VA, USA) diluted with PBS one hour prior to euthanasia. All mice were in diestrus at the time of euthanasia to control cyclic changes in ovarian hormones. Euthanasia was performed by CO_2_ asphyxiation. The right inguinal mammary gland was collected and fixed in 10% neutral buffered formalin (5701, Epredia, Kalamazoo, MI, USA) for 48 h, then embedded in paraffin. The left inguinal and thoracic mammary glands were collected and digested for one hour at 37 °C in Dulbecco’s Modified Eagle Medium (DMEM, 10-017-CV, Corning, Corning, NY, USA) supplemented with 10% fetal bovine serum (FBS, A52567-01, Gibco, Grand Island, NE, USA), 1% Antibiotic–Antimycotic Solution (30-004-CI, Corning), 10 µg/mL insulin (I0516, Sigma-Aldrich), 5 ng/mL human epidermal growth factor (E9644, Sigma-Aldrich), 0.5 µg/mL hydrocortisone (H0888, Sigma-Aldrich), 3 mg/mL collagenase A (11088793001, Sigma-Aldrich), and 100 U/mL hyaluronidase (H3506, Sigma-Aldrich). Digested tissue was cryopreserved for in vitro studies. The liver and visceral fat were collected and weighed, and a section of the liver was snap-frozen for molecular analyses or fixed and embedded in paraffin.

The estrus cycle was tracked using vaginal cytology for 14 days prior to euthanasia. Vaginal epithelial cells were collected by washing the vaginal opening with 50 µL phosphate-buffer saline (PBS) and placed on microscope slides (22-034-979, Fisherbrand, Fisher Scientific, Hampton, NH, USA). The slides were then stained with Wright’s Stain (9350-16, Ricca Chemical Company, Batesville, IN, USA), and the stage of the estrus cycle was assessed using light microscopy as described [51].

### 2.2. Mammary Gland Dissociation

Digested mammary tissues were plated for 1 h in DMEM supplemented with 10% FBS, 1% Antibiotic–Antimycotic Solution, 10 µg/mL insulin, 5 ng/mL human epidermal growth factor, and 0.5 µg/mL hydrocortisone to separate the stromal vascular fraction and epithelial organoids. After one hour, the organoids were removed and pelleted. Cells were washed with 1× PBS, disrupted using a 20 g needle (14817209, Fisher Scientific), and incubated with 2 mL of 0.25% trypsin/ethylenediaminetetraacetic acid (EDTA, 25-053-CI, Corning) for 10 min in a 37 °C water bath. Trypsin was quenched with media containing FBS and 0.1 mg/mL DNase I (10104159001, Sigma-Aldrich), filtered with a 40 µm cell strainer (352340, Falcon, Fisher Scientific), and counted with a hemocytometer (3200, Hausser Scientific, Horsham, UK).

### 2.3. Immunohistochemistry and Immunofluorescence

Paraffin-embedded tissues were sectioned by the Experimental Pathology Laboratory (Carbone Cancer Center, University of Wisconsin–Madison) and then stained with hematoxylin and eosin (H&E) to quantify mammary adipocyte diameters and lipid droplets in the liver. Tissues were stained as described [17]. Slides were blocked in 1% bovine serum albumin (BSA) in tris-buffered saline and Tween-20 (TBST), 5% fish gel in TBST, or using the MOM kit (BMK-2202, Vector Labs, Newark, NJ, USA) for one hour. Primary antibodies were incubated overnight at 4 °C and included cleaved caspase-3 (1:400; 9661T, Cell Signaling, Danvers, MA, USA), BrdU (1:50; OBT0030S, Accurate Chemical & Scientific Corp, Carle Place, NY, USA), F4/80 (1:100; 123102, BioLegend, San Diego, CA, USA), and 8-hydroxy-2′deoxyguanosine (OHdG, 1:100; 501015749, Fisher Scientific). The secondary antibodies were biotinylated goat anti-rabbit (1:500; BA-1000, Vector Labs), biotinylated rabbit anti-rat (1:400; BA-4000, Vector Labs), biotinylated horse anti-mouse (1:500; 30044, Vector Labs), Alexa Fluor 488 goat anti-rabbit H + L (1:250; A11008, Fisher Scientific), and Alexa Fluor 546 goat anti-rat H + L (1:250; A11081, Fisher Scientific). Biotinylated secondary antibodies were visualized with ImmPACT DAB Peroxidase Substrate Kit (SK-4105, Vector Labs). For immunofluorescent stains, nuclei were counterstained with 4′,6-diamidino-2-phenylindole (DAPI). All tissues were imaged on the Nikon Eclipse E600. All images were quantified using ImageJ v 1.52a (National Institutes of Health, Staten Island, NY, USA). Five ducts per tissue section were imaged from five mice per group. Positive and negative cells were counted in the ducts, and the number of positive cells was divided by the total number of cells and multiplied by 100. F4/80 staining was quantified by taking five images per tissue section from five mice per group, and the number of crown-like structures within the field of view was quantified.

### 2.4. Cell Culture and Transfection Experiments

COMMA-D mammary epithelial cells were provided by Dr. Charlotte Kuperwasser (Tufts University, Boston, MA, USA). A total of 293T cells were obtained from American Type Culture Collection (CRL-3216, Manassas, VA, USA). All cells were cultured in DMEM supplemented with 10% FBS and 1% Antibiotic–Antimycotic Solution at 37 °C at 5% CO_2_. COMMA-D cells were treated with 9.8 µM acrylamide, 0.5 mM glycidamide, 100 µM hydrogen peroxide, or vehicle for 24 h, based on the literature of treatment of cells with acrylamide or glycidamide in other contexts [39,52,53,54,55].

Bacterial stocks of *Cyp2e1* shRNA (TRCN0000011869, MilliporeSigma, Burlington, MA, USA) and human *Cyp2e1* (HsCD00942861, DNASU Plasmid Repository, Tempe, AZ, USA) were grown for 2 days on agar with 25 mg/mL ampicillin (BP90225, Fisher Scientific) in a 37 °C incubator. Single colonies were isolated and expanded for 8 h in 1 mL lysogeny broth (LB) medium, then 100 µL of that stock was further expanded overnight in 100 mL LB medium at 37 °C in a shaker. Plasmids were extracted using the Invitrogen PureLink HiPure Plasmid Filter Maxiprep Kit (K210016). Three µg of isolated plasmid, 2 µg pCMV ΔR 8.2 Δvpr, and 1 µg pCMV-VSVG plasmids were transfected into 293T cells with *Trans*IT-2020 (MIR 5400, Mirus Bio, Madison, WI, USA). Transduced 293T cells were grown for 24 h; then, the media were removed and filtered with 0.45 µm syringe filters (09-720-005, Fisherbrand, Fisher Scientific) and incubated with COMMA-D cells. COMMA-D cells were selected with 40 µg/mL puromycin (AAJ67236XF, Fisher Scientific) or 10 µg/mL blasticidin (R21001, Fisher Scientific) for 5 days.

### 2.5. Immunocytochemistry

COMMA-D cells were plated on 8-well chamber slides (Falcon, 354118), treated for 24 h after reaching confluence, and fixed with 100% ice-cold methanol for 10 min at −20 °C. Cells were permeabilized with 0.1% Triton-X in PBS. The slides were either blocked in 1% BSA in TBST for one hour at room temperature or incubated with 2 M HCl for one hour at room temperature prior to labeling for BrdU. Primary antibodies included 8-hydroxydeoxyguanosine (8-OHG, 1:200; NB600-1508, Novus Biologicals, Centennial, CO, USA), phosphorylated histone H2AX (γH2AX, 1:100), and BrdU (1:50). The secondary antibodies included Alexa Fluor 546 donkey anti-goat H + L (Invitrogen, Waltham, MA, USA, A11056), Alexa Fluor 488 goat anti-rabbit H + L, and Alexa Fluor 546 goat anti-rat H + L. Nuclei were counterstained with DAPI and imaged with the Nikon Eclipse E600 (Nikon Instruments, Inc., Melville, NY, USA). Three experiments were plated in duplicate, and five images per well were taken. The mean cell intensity was quantified using ImageJ.

### 2.6. Alkaline Comet and FLARE Assays

Alkaline comet assays were performed in triplicate following the Trevigen CometAssay HT (4252-040-K) protocol with some modifications. Five hundred mammary epithelial cells or COMMA-D cells were combined with 50 µL of CometAssay LMAgarose (4250-200-03, R&D Systems, Minneapolis, MI, USA) and spread on CometSlides (4250-200-03, R&D Systems) pre-warmed to 37 °C. After the agarose gels solidified for 30 min at 4 °C, slides were immersed in lysis buffer (4250-050-K, R&D Systems) overnight at 4 °C. Slides were then transferred to Alkaline Unwinding Solution (200 mM NaOH; 1 mM EDTA) for one hour at room temperature in the dark. Gel electrophoresis was performed in Alkaline Electrophoresis Solution (300 mM NaOH, 1 mM EDTA) for 40 min at 25 V at 4 °C. Cells were stained with 100 µL SYBR Gold Nucleic Acid Gel Stain (S11494, Fisher Scientific) for 30 min at room temperature. Fifty to sixty cells from each slide were imaged.

Fragment Length Analysis using Repair Enzyme (FLARE) assays with the formamidopyrimidine DNA glycosylase (FPG) enzyme were performed in triplicate under neutral conditions following the Trevigen CometAssay HT (4252-040-K) protocol with some modifications. COMMA-D cells were plated and lysed overnight, as described above. Slides were then transferred to Enzyme Buffer (40 mM HEPES, 0.5 mM EDTA, 0.1 M KCl, pH7.6) for one hour at 4 °C. FPG was added at 1:1000 to pre-warmed Enzyme Buffer with 0.2 mg/mL BSA. A total of 100 µL of FPG Enzyme Buffer was added to each sample gel and incubated for one hour at 37 °C in a humidified chamber. Control gels received Enzyme Buffer with BSA without FPG. Slides were transferred to 1× Neutral Electrophoresis Buffer (0.05 M Tris Base, 0.15 M Sodium Acetate) for 30 min at 4 °C then gel electrophoresis was performed in 1× Neutral Electrophoresis Buffer at 25 V for 40 min at 4 °C. After incubation in DNA Precipitation Solution (1 M Ammonium Acetate in 95% EtOH), fixation with 70% EtOH, and drying for 15 min at 37 °C, cells were stained with SYBR Gold. Twenty-five to thirty-five cells from each slide were imaged. All slides from the alkaline comet assays and FLARE assays were imaged with Nikon Eclipse E600 and analyzed on ImageJ with OpenComet v1.3.1(https://cometbio.org/, accessed on 28 April 2024).

### 2.7. ROS Assay

ROS were measured following the protocol provided by CellROX Green Reagent Kit (C10444, Fisher Scientific). Briefly, COMMA-D cells were cultured on a 96-well plate until confluence occurred, and then they were treated. After 24 h, cells were washed with 1× PBS and incubated with 5 µM/well CellROX Green for 30 min at 37 °C. Wells were washed three times with PBS and fixed in 3.7% formaldehyde, and nuclei were counterstained with DAPI. Cells were imaged with the Keyence BZ-X710 (Keyence, Itasca, IL, USA), and images were quantified by measuring fluorescent intensity divided by DAPI intensity using ImageJ.

### 2.8. Western Blots

Protein was extracted from tissues and COMMA-D cells using Radio-Immunoprecipitation Assay (RIPA) buffer and Protease Inhibitor Cocktail (G651, Promega, Madison, WI, USA). Isolated protein was loaded into 10% SDS Page gels (4568034, Bio-Rad, Hercules, Rome, Italy) at 20 µg/well. Electrophoresis was performed at 150 V in 10% Tris/Glycine/SDS Buffer (1610732, Bio-Rad). Protein was transferred from the gel to a nitrocellulose membrane (RPN303D, GE Healthcare, Madison, WI, USA) in 10% Tris/Glycine Buffer (1610771, Bio-Rad) at 100 V for one hour. Protein transfer was confirmed with Ponceau S staining (P7170, Sigma-Aldrich). The nitrocellulose membrane was washed with 0.1% TBST until Ponceau staining cleared and was blocked with 5% milk in 0.1% TBST for one hour at room temperature on an orbital shaker. Membranes were then probed with either anti-CYP2E1 (1:500; 19937-1-AP, Fisher Scientific), or anti-glyceraldehyde 3-phosphate dehydrogenase (GAPDH, 1:10,000; MA5-15738, Fisher Scientific) overnight at 4 °C on an orbital shaker. After washing with 0.1% TBST, membranes were probed with secondary goat anti-rabbit (1:10,000; 31460, Fisher Scientific) or anti-mouse (1:10,000; 31430, Fisher Scientific) conjugated to horseradish peroxidase (HRP) for one hour at room temperature on an orbital shaker. Membranes were treated with SuperSignal West Femto Maximum Sensitivity Substrate (34095, Fisher Scientific,) for five minutes, then the signal was detected using film (F-9023, GeneMate, VWR International, Batavia, IL, USA) developed on the All-Pro Imaging Corp 100 Plus Automatic X-Ray Film Processor (Melville, NY, USA). Protein content was quantified by measuring pixels on ImageJ.

### 2.9. Quantitative Real-Time Polymerase Chain Reaction

RNA was extracted with TRIzol (15596018, Fisher Scientific) and PureLink RNA Mini Kit (12183018A, Fisher Scientific) or with the Quick-DNA/RNA Miniprep Kit (D7001, Zymo Research, VWR International, Radnor, PA, USA). The RNA was reverse transcribed using the High-Capacity cDNA Reverse Transcription Kit (4368814, Applied Biosystems, Fisher Scientific) and Biometra Thermal Cycler (Analytik Jena, Jena, Germany). Quantitative PCR was performed with iTaq Universal SYBR Green Supermix (1725121, Bio-Rad) on a Bio-Rad CFX Connect Real-Time PCR Detection System (Bio-Rad). Data were analyzed using the ΔCT or the ΔΔCT method. Transcripts were normalized to hypoxanthine phosphoribosyl transferase (HPRT). Primer sequences are found in Table S1.

### 2.10. Glutathione Assay

Glutathione and oxidized glutathione dimers (GSSG) were measured using the Glutathione Colorimetric Detection kit (Fisher Scientific, EIAGSHC). COMMA-D cells were lysed in 5% 5-sulfosalicylic acid dihydrate, and samples were diluted 1:5 in assay buffer. GSSG was measured by treating samples and standards with 2-vinylpyridine. Absorbance was read at 405 nm, and cellular concentrations of total glutathione and GSSG were calculated from the respective standard curves.

### 2.11. Statistical Analysis

Results are reported as the mean ± standard error of the mean (s.e.m.). Statistical differences were determined using one-way analysis of variance (ANOVA) and Tukey’s multiple comparisons post-test unless otherwise noted. A *p*-value of ≤0.05 denotes significant value. All statistical analyses were performed with GraphPad Prism 9.4.1 (GraphPad Software, La Jolla, CA, USA).

## 3. Results

### 3.1. Acrylamide Does Not Enhance Obesity-Associated Changes to the Mammary Gland

**Figure 1:**
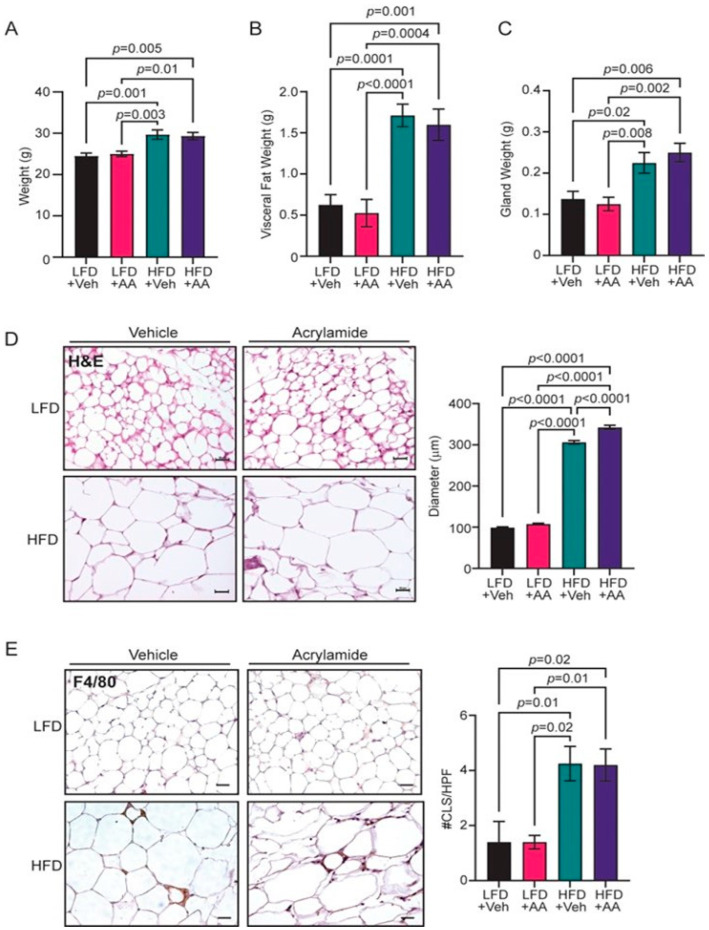
Acrylamide does not enhance weight gain. (**A**) Body weights of FVB/N female mice fed LFD or HFD and treated with vehicle (Veh) or 0.7 mM acrylamide (AA) water for 16 weeks (*n* = 6–8 mice/group). (**B**) Visceral fat weight after 16 weeks of treatment (*n* = 6–8 mice/group). (**C**) Mammary gland weights after 16 weeks of treatment (*n* = 6–8 mice/group). (**D**) Representative images and quantification of mammary adipocyte diameters (*n* = 6–8 mice/group). (**E**) Representative images and quantification of F4/80^+^ crown-like structures (CLS) per high power field (HPF; *n* = 5 mice/group). Bars represent mean ± s.e.m. Magnification bars = 50 µm.

To examine how acrylamide impacts the mammary gland under conditions of obesity, FVB/N female mice received LFD or HFD and water or 0.7 mM acrylamide-supplemented water ad libitum for 16 weeks. Chronic treatment of mice and rats with acrylamide at this concentration was found to promote mammary tumorigenesis [56]. No differences were observed in the water consumption in mice in any of the groups (Figure S1A). After 16 weeks, mice fed HFD were significantly heavier than mice fed LFD (*p* = 0.001, Figure 1A). However, acrylamide treatment had no impact on final body weights (Figure 1A) or weight gain (Figure S1B) compared to the respective controls. In addition to body weights, HFD-fed mice had significantly increased visceral fat weights (*p* = 0.0001, Figure 1B) and mammary gland weights (*p* = 0.02, Figure 1C), but acrylamide-treated mice did not significantly differ in either endpoint compared to vehicle-treated mice on each respective diet (Figure 1B,C). Consistent with elevated mammary gland mass, HFD-fed mice had increased adipocyte diameters compared to LFD-fed mice (*p* < 0.0001, Figure 1D). Acrylamide treatment of LFD-fed mice did not alter adipocyte diameters compared to control LFD-fed mice (Figure 1D). In contrast, acrylamide treatment of HFD-fed mice had a further significant increase in adipocyte diameters compared to HFD-fed mice (*p* < 0.0001, Figure 1D). A hallmark of obesity is the formation of crown-like structures, which are formed when F4/80^+^ macrophages are recruited into the mammary gland and surround dying adipocytes [57]. F4/80^+^ crown-like structures were significantly elevated in the mammary glands of HFD-fed mice compared to LFD-fed mice (*p* = 0.01, Figure 1E). However, acrylamide treatment did not further enhance crown-like structures in the mammary glands compared to their respective controls (Figure 1E). Taken together, these results show that HFD-induced obesity and the addition of acrylamide treatment further increased adipocyte diameters, but acrylamide did not significantly increase weight gain or inflammation.

### 3.2. Acrylamide Induces Systemic Changes in CYP2E1 Expression and DNA Damage

**Figure 2:**
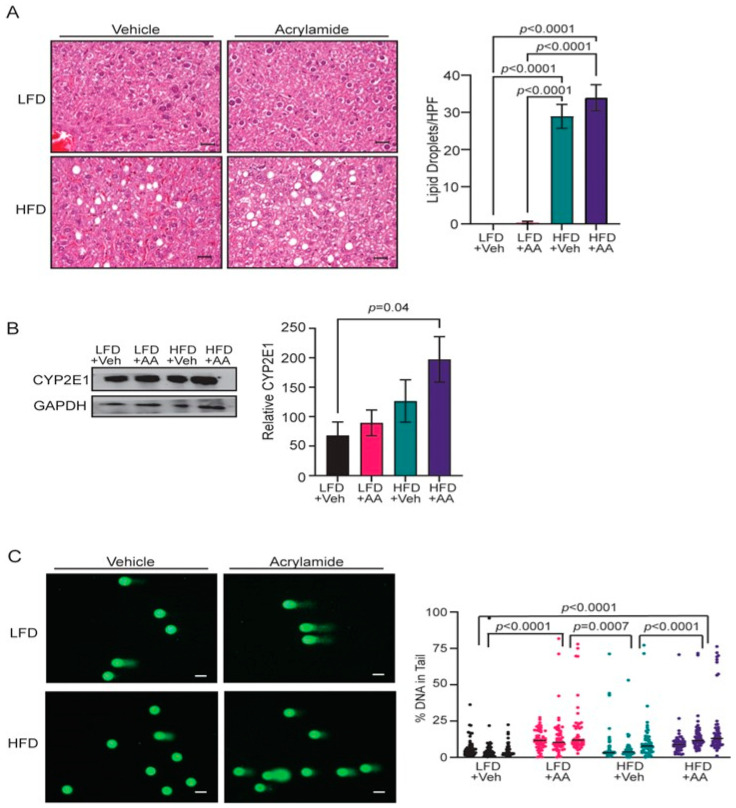
Acrylamide and obesity have systemic effects in mice. (**A**) Representative images and quantification of lipid droplets in H&E-stained sections of liver per HPF from LFD- and HFD-fed mice with and without AA treatment (*n* = 5 mice/group). (**B**) Western blot and quantification of CYP2E1 protein from the livers relative to GAPDH (*n* = 5 mice/group). (**C**) Representative images of the alkaline comet assay of PBMCs and quantification of the percentage of DNA in comet tails (*n* = 50–60 cells/mouse, 3 mice/group). Bars represent mean ± s.e.m. Magnification bars = 50 µm.

The liver is a site of ectopic lipid deposition in obesity, and acrylamide exposure could enhance lipid in this tissue. Liver weights were not significantly different in HFD-fed mice compared to LFD-fed mice, and acrylamide treatment did not enhance liver weights compared to controls (Figure S1C). Although not severe enough to impact liver weight, HFD-fed mice had significantly more fat droplets in the liver than LFD-fed mice (*p* < 0.0001, Figure 2A). However, acrylamide-treatment did not increase lipid droplets in the liver of either obese or lean mice, suggesting acrylamide does not enhance fat storage in the liver to elevate the negative effects of obesity.

Mammary gland development and function are regulated by the ovarian steroids estrogen and progesterone [58]. To examine how exposure to HFD or acrylamide altered ovarian function, vaginal cytology was performed for two weeks to identify changes in the estrus cycle. As shown previously [17], exposure to HFD did not significantly alter time spent in either estrus or diestrus compared to LFD-fed mice (Figure S1D,E). Similarly, acrylamide-treated mice did not differ in the number of days spent in estrus or diestrus compared to control vehicle-treated mice (Figure S1D,E).

Recent studies suggest that individuals with obesity have enhanced activity of the acrylamide-metabolizing P450 enzyme, CYP2E1, in the liver [59]. The relative expression of *Cyp2e1* in the liver was not significantly different among mice in any of the groups, although levels were mildly elevated in the obese, acrylamide-treated group (Figure S1F). However, HFD-fed mice supplemented with acrylamide had significantly higher levels of CYP2E1 protein in the liver compared to vehicle-treated LFD-fed mice (*p* = 0.04, Figure 2B). These results suggest that obesity and acrylamide together increase the levels of CYP2E1 protein in the liver. Elevated levels of CYP2E1 may increase the metabolism of acrylamide to the genotoxic metabolite glycidamide. To test this hypothesis, DNA damage in peripheral blood mononuclear cells (PBMC) was quantified using alkaline comet assays. No difference in DNA damage was observed in PBMC isolated from LFD and HFD-fed mice (Figure 2C). However, PBMCs isolated from both LFD- and HFD-fed acrylamide-treated mice demonstrated elevated DNA damage compared to both groups of control mice (Figure 2C). The DNA damage present in circulating cells suggests that exposure to acrylamide can induce DNA damage systemically, potentially via glycidamide.

### 3.3. Acrylamide Exacerbates DNA Damage and Apoptosis in Mammary Glands from Obese Mice

**Figure 3:**
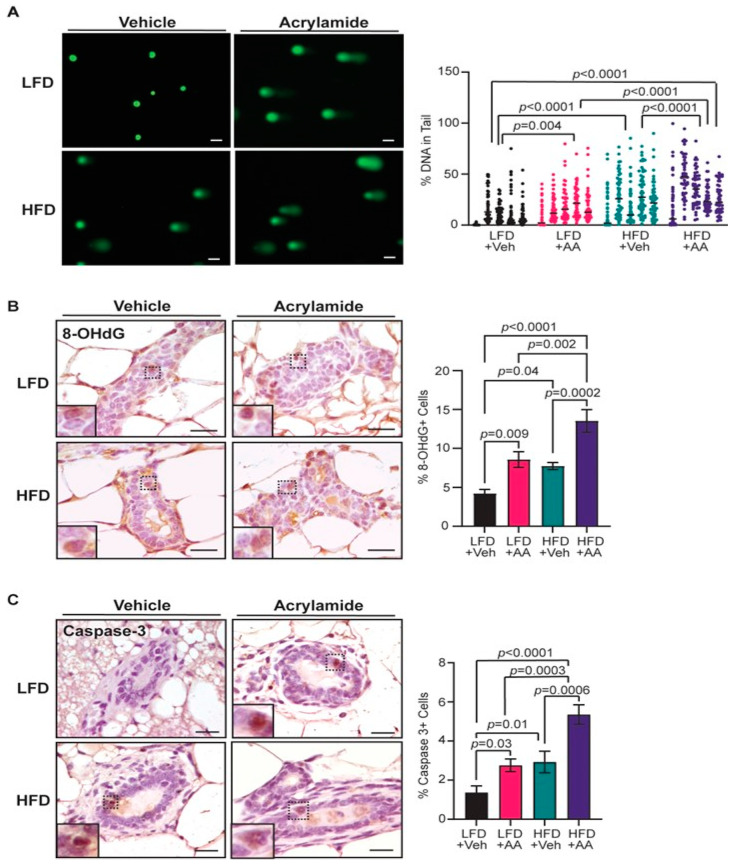
Acrylamide treatment enhances DNA damage and apoptosis in the mammary glands of obese mice. (**A**) Representative images of alkaline comet assays of mammary epithelial cells and quantification of the percentage of DNA in comet tails (*n* = 50–60 cells/mouse, 5 mice/group). (**B**) Representative images and quantification of the percentage of 8-OHdG^+^ cells in mammary ducts (*n* = 5 mice/group). (**C**) Representative images and quantification of the percentage of cleaved caspase 3^+^ cells in mammary ducts (*n* = 5 mice/group). Bars represent mean ± s.e.m. Magnification bars = 50 µm.

To test how long-term exposure to acrylamide impacts DNA damage in mammary epithelial cells, alkaline comet assays were performed on mammary epithelial cells isolated from mice from each group. HFD-fed mice had significantly elevated DNA damage compared to LFD-fed mice (*p* < 0.0001, Figure 3A). LFD-fed mice treated with acrylamide also had significantly more DNA damage compared to lean mice alone (*p* = 0.004, Figure 3A), with comparable levels of DNA damage as control HFD-fed mice. Acrylamide-treated HFD-fed mice had significantly more DNA damage compared to all three other groups (Figure 3A), suggesting that obesity and acrylamide together enhance mammary epithelial DNA damage.

Oxidative DNA adducts are a form of DNA damage that can promote DNA–protein crosslinks and stall transcription and replication, and it can act as a mutagen [60]. 8-OHdG is one of the most common oxidative adducts and is highly mutagenic [61]. Mammary ducts from HFD-fed mice had significantly increased 8-OHdG^+^ cells compared to LFD-fed mice (*p* = 0.04, Figure 3B). LFD-fed mice treated with acrylamide also had significantly more 8-OHdG^+^ cells compared to LFD-fed mice without acrylamide treatment (*p* = 0.009, Figure 3B). Mammary ducts from HFD-fed, acrylamide-treated mice had the highest number of 8-OHdG^+^ epithelial cells compared to all other groups (Figure 3B). These results suggest that obesity and acrylamide enhance oxidative DNA damage in mammary epithelial cells.

Due to the enhanced oxidative DNA damage, an analysis of antioxidant levels impacted by acrylamide treatment and diet was conducted. Levels of catalase (*Cat*) and nitric oxide synthase 2 (*Nos2*) were mildly elevated in HFD-fed, acrylamide-treated mice (Figure S2A,B). Levels of superoxide dismutase (*Sod1*) did not differ across treatment groups (Figure S2C). These data suggest that acrylamide exposure did not significantly alter antioxidant expression in mammary epithelial cells.

As elevated levels of DNA damage can lead to increased apoptosis [62], cleaved caspase-3 in mammary epithelial cells was quantified. HFD-fed mice had enhanced levels of cleaved caspase-3 compared to LFD-fed mice (*p* = 0.01, Figure 3C). Additionally, epithelial cells from LFD-fed acrylamide-treated mice had elevated cleaved caspase-3 compared to lean, vehicle-treated mice (*p* = 0.03, Figure 3C). HFD-fed, acrylamide-treated mice had the most cleaved caspase-3^+^ epithelial cells compared to all other groups (Figure 3C). These results suggest that obesity and acrylamide exposure enhance mammary epithelial DNA damage and apoptosis.

### 3.4. Glycidamide Increases Single- and Double-Strand DNA Breaks, but Acrylamide Enhances Oxidative Stress and Oxidative DNA Damage in COMMA-D Cells

**Figure 4:**
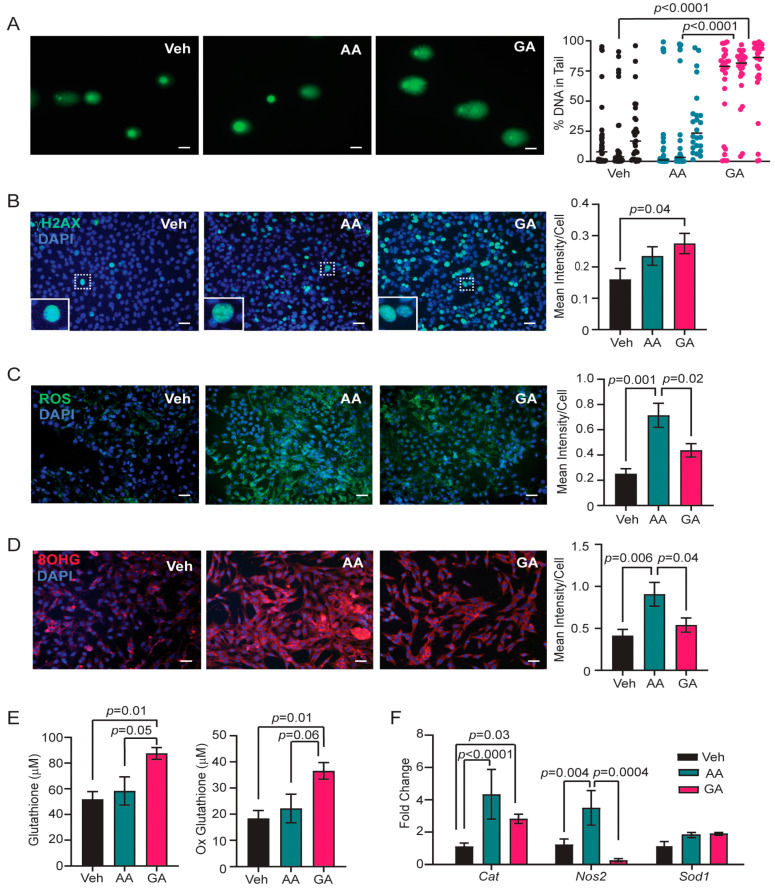
Glycidamide increases DNA breaks, while acrylamide enhances oxidative stress in COMMA-D cells. (**A**) Representative images of the alkaline comet assay and quantification of the percentage of DNA in comet tails of COMMA-D cells treated with vehicle (Veh), 9.8 µM acrylamide (AA), or 0.5 mM glycidamide (GA) (*n* = 25–35 cells/group/replicate). (**B**) Representative images and quantification of the fluorescent intensity of γH2AX^+^ cells divided by total DAPI^+^ cells in COMMA-D cells (*n* = 3 images/group/replicate). (**C**) Representative images and quantification of ROS in Veh, AA, and GA treated cells (*n* = 3 images/well, 2–3 wells/group). (**D**) Representative images and quantification of the fluorescent intensity of 8-OHG^+^ cells divided by total DAPI^+^ cells (*n* = 5 images/group/replicate). (**E**) Glutathione and oxidized glutathione levels in cell lysates of COMMA-D cells treated with Veh, AA, and GA (*n* = 6 samples/group). (**F**) Fold change of *Cat*, *Nos2*, and *Sod1* mRNA relative to *Hprt* in COMMA-D cells (*n* = 3 replicates/group). Bars represent mean ± s.e.m. Magnification bars = 50 µm.

To understand the mechanism of DNA damage in mammary epithelial cells due to acrylamide exposure, COMMA-D epithelial cells were cultured and treated with 9.8 µM acrylamide, 0.5 mM glycidamide, or vehicle for 24 h. These doses were chosen based on the recent literature describing the effects of acrylamide and glycidamide in other contexts [39,52,53,54,55]. No differences in cell viability were observed following treatment with either acrylamide or glycidamide compared to vehicle-treated cells. COMMA-D cells were also treated with 100 µM hydrogen peroxide as a positive control for DNA damage. Hydrogen peroxide treatment enhanced levels of DNA strand breaks measured by alkaline comet assays (*p* < 0.0001, Figure S3A). When COMMA-D cells were treated with acrylamide, elevated DNA strand breaks were not observed when measured by the comet assays (Figure 4A). However, glycidamide treatment significantly increased DNA damage within the cells compared to both vehicle and acrylamide treatment (Figure 4A). Hydrogen peroxide treatment also enhanced the expression of γH2AX (*p* = 0.002, Figure S3B). In contrast, γH2AX levels were not different between vehicle and acrylamide treatment (Figure 4B). Glycidamide treatment increased γH2AX levels compared to vehicle-treated cells (*p* = 0.04, Figure 4B). These results suggest glycidamide, rather than acrylamide, increases DNA strand breaks.

To assess if acrylamide or glycidamide enhances oxidative stress in COMMA-D cells, levels of intracellular ROS were measured. Hydrogen peroxide treatment enhanced ROS (*p* < 0.0001, Figure S3C). Surprisingly, ROS levels were significantly higher in acrylamide-treated cells than in either vehicle or glycidamide-treated cells (Figure 4C). Elevated oxidative stress can also result in the formation of oxidative adducts on RNA in addition to adducts on DNA. COMMA-D cells were examined for the oxidative RNA adduct 8-OHG. Hydrogen peroxide treatment did not significantly increase 8-OHG levels compared to the vehicle (Figure S3D). However, acrylamide treatment significantly increased 8-OHG adducts compared to both vehicle and glycidamide treatment (Figure 4D), suggesting that acrylamide elevates levels of oxidative stress. Together, this suggests acrylamide enhanced oxidative stress via increased ROS levels within cells, leading to damaged cytosolic RNA.

Glutathione is one of the main antioxidants involved in cellular ROS detoxification. Glutathione is oxidized in response to ROS or can be directly conjugated to acrylamide to promote excretion of acrylamide. Glutathione and oxidized glutathione levels may be depleted in acrylamide-treated cells. Surprisingly, glutathione and oxidized glutathione levels were elevated in glycidamide-treated cells compared to both vehicle- and acrylamide-treated cells (Figure 4E). However, acrylamide treatment led to elevated expression levels of other antioxidants, specifically *Cat* and *Nos2* but not *Sod1* (Figure 4F). Overall, these data indicate that glycidamide enhanced DNA strand breaks, but acrylamide treatment elevated oxidative stress and oxidative RNA damage in COMMA-D cells.

### 3.5. Knockdown of CYP2E1 in COMMA-D Cells Rescues Acrylamide-Induced Oxidative Stress and Oxidative DNA Damage

**Figure 5:**
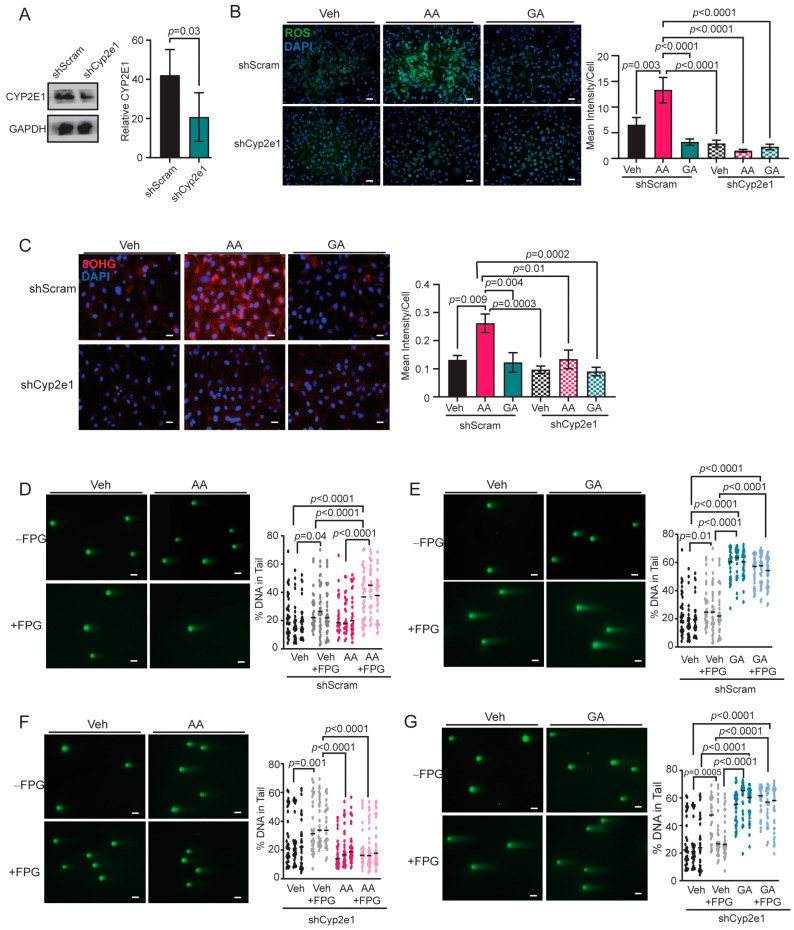
Loss of CYP2E1 reduces oxidative stress in COMMA-D cells. (**A**) Western blot and quantification of CYP2E1 protein relative to GAPDH in scrambled shRNA (shScram) and shCyp2e1 COMMA-D cells (*n* = 3 replicates). Significance was determined using a paired Student’s *t*-test. (**B**) Representative images and quantification of cellular ROS in shScram or shCyp2e1 COMMA-D cells treated with Veh, AA, or GA (*n* = 3 images/well, 2 wells/group). (**C**) Representative images and quantification of fluorescent intensity of 8-OHG^+^ cells divided by total DAPI^+^ cells in shScram or shCyp2e1 COMMA-D cells (*n* = 5 images/well, 3 wells/group). (**D**) Representative images of the neutral FLARE assay (+FPG indicates the addition of formamidopyrimidine DNA glycosylase (FPG) enzyme) and quantification of the percentage of DNA in comet tails of shScram COMMA-D cells treated with Veh or AA (*n* = 25–35 cells/group/replicate). (**E**) Representative images of the neutral FLARE assay and quantification of the percentage of DNA in comet tails of shScram COMMA-D cells treated with Veh or GA (*n* = 75–95 cells/group). (**F**) Representative images of the neutral FLARE assay and quantification of the percentage of DNA in comet tails of shCyp2e1 COMMA-D cells treated with Veh or AA (*n* = 75–95 cells/group). (**G**) Representative images of the neutral FLARE assay and quantification of the percentage of DNA in comet tails of shCyp2e1 COMMA-D cells treated with Veh or GA (*n* = 75–95 cells/group). Bars represent mean ± s.e.m. Magnification bars = 50 µm.

The activation of CYP2E1 has been shown to increase cellular oxidative stress [63,64]. While CYP2E1 is highly expressed in the liver (Figure S4A), we observed that CYP2E1 protein was also present in COMMA-D cells (Figure S4A), and *Cyp2e1* transcripts were detected in primary mammary epithelial cells isolated from mice in all treatment groups (Figure S4B). Acrylamide-associated oxidative stress may be due to the activation of CYP2E1 within epithelial cells. COMMA-D cells were transduced with lentivirus encoding either shRNA scrambled control (shScram) or shRNA targeting *Cyp2e1* (shCyp2e1) as well as lentivirus to overexpress *Cyp2e1* to generate stable cell lines. Compared to shScram cells, shCyp2e1 cells had significantly reduced CYP2E1 protein (*p* = 0.03, Figure 5A), while overexpressing cells had elevated CYP2E1 expression compared to controls (*p* = 0.006, Figure S4C). When shScram cells were treated with acrylamide, elevated intracellular ROS levels were observed compared to both vehicle- and glycidamide-treated shScram cells (Figure 5B). However, treatment of shCyp2e1 cells with acrylamide did not elevate ROS levels compared to either shScram cells treated with acrylamide or shCyp2e1 cells treated with vehicle or glycidamide (Figure 5B). These data demonstrate that loss of CYP2E1 prevented acrylamide-induced increases in cellular ROS. In contrast, the acrylamide treatment of overexpressing CYP2E1 cells significantly increased intracellular ROS levels compared to overexpressing CYP2E1 cells treated with either vehicle or glycidamide (Figure S4D). Further, acrylamide-treated, CYP2E1 overexpressing cells had significantly elevated ROS compared to all treatments in control cells (Figure S4D). These data support a CYP2E1-driven increase in ROS in COMMA-D cells in response to acrylamide treatment.

Levels of oxidative RNA adduct 8-OHG were examined. In shScram cells, acrylamide treatment significantly increased 8-OHG RNA adducts compared to shScram cells treated with vehicle and glycidamide (Figure 5C). However, the treatment of shCyp2e1 cells with acrylamide did not enhance 8-OHG adducts compared to vehicle or glycidamide-treated cells (Figure 5C). Further, the overexpression of CYP2E1 resulted in enhanced levels of 8-OHG adducts after acrylamide treatment compared to all treatments of control cells and overexpressing CYP2E1 cells treated with the vehicle or glycidamide (Figure S4E). These results support the hypothesis that acrylamide-driven oxidative adducts are a result of CYP2E1 activation.

Oxidative DNA damage can also be assessed utilizing a modified comet assay called the fragment length analysis using restriction enzymes (FLARE) assay. This assay includes treatment of permeabilized cells with FPG enzyme, which excises oxidized DNA bases to create single strand DNA breaks detectable by electrophoresis. These DNA breaks would otherwise go undetected by an unmodified comet assay. Acrylamide treatment may increase oxidative DNA damage due to elevated oxidative stress. In shScram cells, increased DNA damage was present in vehicle-treated cells with FPG (*p* = 0.04, Figure 5D), indicating basal levels of oxidative DNA damage in these cells due to cell culture conditions. Consistent with our previous observation in comet assays (Figure 4A), no differences in DNA damage were detected in shScram cells treated with acrylamide without FPG compared to vehicle-treated cells without FPG (Figure 5D). However, FPG treatment revealed that acrylamide-treated shScram cells had significantly increased DNA damage compared to non-FPG, acrylamide-treated cells (*p* < 0.0001) as well as cells that were treated with vehicle and vehicle with FPG (Figure 5D). When shScram cells were treated with glycidamide, increased DNA damage was present compared to vehicle-treated cells (Figure 5E). However, treatment of cells with FPG did not further enhance the levels of DNA damage (Figure 5E). These data indicate that glycidamide treatment does not induce oxidative DNA damage.

The loss of CYP2E1 expression and its impact on oxidative DNA damage was measured with FLARE assays. Similar to shScram cells (Figure 5D), vehicle-treated shCyp2e1 cells showed elevated DNA damage when treated with FPG compared to without FPG (*p* = 0.001, Figure 5F). When shCyp2e1 cells were treated with acrylamide, DNA damage was not different compared to vehicle-treated cells either with or without the addition of FPG (Figure 5F), showing that loss of CYP2E1 eliminated oxidative DNA damage due to inhibition of phase I acrylamide metabolism. In contrast, glycidamide-treated shCyp2e1 cells had significantly increased DNA damage compared to shCyp2e1 vehicle-treated cells with and without FPG (*p* < 0.0001, Figure 5G), but the addition of FPG to glycidamide-treated cells did not further increase DNA damage (Figure 5G). Overall, these data suggest acrylamide but not glycidamide enhances oxidative DNA damage mediated by CYP2E1.

## 4. Discussion

Obesity and carcinogen exposure both increase the risk for breast cancer [3,4,12,13], but there is limited knowledge on how obesity and carcinogens interact to promote breast cancer risk. Acrylamide has been suggested to be both a carcinogen and an obesity-inducing agent [26,33,34], and these studies addressed the knowledge gap on the effects of acrylamide exposure on weight gain and DNA damage in mammary epithelial cells under conditions of obesity. Our results suggest that exposure to acrylamide at a dose similar to human exposure has limited effects as an obesity-inducing agent but enhances mammary epithelial DNA damage, which may increase the risk of breast cancer. These results are significant due to the high prevalence of acrylamide in the Western diet [28,29]. While CYP2E1 is expressed within the liver, CYP2E1 expression was also identified in mammary epithelial cells, suggesting that acrylamide is metabolized within the mammary gland as well. The metabolism of acrylamide by CYP2E1 led to elevated epithelial oxidative stress and increased DNA damage. Elevated levels of acrylamide consumption through the Western diet could promote genotoxic effects through both ROS generation and glycidamide exposure within mammary epithelial cells. Consistent with studies focused on patients with obesity [59], obese mice had elevated levels of CYP2E1. Increased obesity through the Western diet may further fuel DNA damage through elevated expression of CYP2E1 to metabolize acrylamide to genotoxic intermediates. Additional studies are necessary to identify how obesity and acrylamide exposure alter mammary tumorigenesis.

An obesogen is a compound that can cause both metabolic dysfunction and weight gain [65]. While acrylamide has been shown to enhance weight gain in mouse and zebrafish models [33,34], there is contradictory epidemiological evidence associating acrylamide exposure with elevated weights in humans [66,67,68]. The chronic treatment of mice with acrylamide-supplemented water did not increase body, visceral fat, liver, or mammary gland weights after exposure for 16 weeks in lean or obese mice. These results are in agreement with another study where mice and rats were exposed to acrylamide for two years at the same dose as used in this study, and no changes in body weight were identified [56]. However, Lee et al. reported increased body weight with acrylamide treatment when 50 µg/kg of acrylamide was administered to mice through oral gavage [33]. Acrylamide is rapidly absorbed through the gut and has an elimination half-life of 1–6 h in mice and rats, depending on the tissue type [69,70,71]. It is possible that administration of a large bolus of acrylamide through oral gavage could have different effects on weight gain than continuous administration of lower levels of acrylamide in drinking water. Other studies have demonstrated that higher doses of acrylamide (2–20 mg/kg body weight) did not impact body weight in rats but instead increased serum levels of cholesterol, glucose, and triglycerides [72,73,74]. Elevated levels of cholesterol, triglycerides, and glucose are associated with heart disease and diabetes [75,76], suggesting that acrylamide could act as an obesogen to increase the risk for metabolic diseases at high doses. A limitation of our study is that we did not measure metabolic markers, such as serum triglycerides, in addition to weight gain to investigate both the weight-inducing and metabolic properties of acrylamide. Increased adipocyte diameters in obese, acrylamide-treated mice may indicate acrylamide-associated disruption to lipid metabolism, but more research is needed to elucidate how acrylamide impacts adipocyte metabolism in this model.

The acrylamide metabolite, glycidamide, is thought to be the agent responsible for DNA damage and carcinogenesis after acrylamide exposure rather than acrylamide itself. Glycidamide is genotoxic and forms mutagenic adducts at a faster rate than acrylamide [77]. Glycidamide, rather than acrylamide, induced DNA strand breaks in COMMA-D epithelial cells assessed with comet assays, consistent with literature demonstrating glycidamide induces DNA adducts and subsequent DNA damage [78,79,80]. Acrylamide can form guanine adducts, but they form at a slower rate than glycidamide adducts [77], do not undergo spontaneous depurination to become mutagenic [77,81], and, therefore, have fewer biological consequences [77]. DNA damage in epithelial cells from obese, acrylamide-treated mice was elevated compared to all other groups. Elevated levels of CYP2E1 protein in the liver likely lead to increased conversion of acrylamide to glycidamide and higher circulating levels of mutagen glycidamide. In contrast, isolated PBMCs from lean and obese mice showed similar levels of DNA damage from acrylamide exposure. This may be in part due to the short lifespan of PBMCs [82]. The glycidamide-induced DNA damage observed in epithelial cells may elevate the risk for cancer. Newborn B6C3F_1_ mice treated with three doses of 0.7 mM/kg glycidamide developed significantly more hepatic mutations and hepatocarcinomas than mice identically treated with vehicle or acrylamide [38]. Further, in mouse embryo fibroblasts with a human-TP53 knock-in gene, treatment with 1–3 mM glycidamide for 24 h created a mutational signature characterized by A > T and T > A point mutations [83,84]. This mutational signature has been found in breast cancer patients and individuals who smoke [83,84]. In contrast, treatment of these cells with 3–10 mM of acrylamide for 48 h did not alter the amount or types of mutations from standard cell culture conditions [83,84]. However, these mouse embryo fibroblasts had a low to no expression of CYP2E1 [83,84], preventing the analysis of oxidative mutational signatures. Future work is necessary to understand how obesity impacts the mutational signatures of glycidamide- and acrylamide-associated oxidative mutations and subsequent mammary tumorigenesis.

A variety of antioxidant pathways may be activated in response to cellular oxidative stress. Marković Filipović et al. demonstrated acrylamide treatment at levels of 25 or 50 mg/kg of body weight for 3 weeks promoted the activation of iNOS, SOD1, and SOD2 antioxidants in the liver [85]; however, the elevated expressions of antioxidants *Cat*, *Nos2*, or *Sod1* in mammary epithelial cells were not observed with acrylamide treatment. Recent work has shown that basal and luminal epithelial cell populations in the mammary gland have different antioxidant capacities [86]. Basal mammary epithelial cells primarily utilize glutathione-dependent mechanisms for antioxidant control, while luminal cells utilize both glutathione-dependent and -independent pathways for antioxidants, including SOD. Basal and luminal cells were not separated during RNA extraction in this study, potentially masking antioxidant responses in specific types of epithelial cells. However, increased levels of glutathione and oxidized glutathione were detected in COMMA-D cells treated with glycidamide. This may indicate a role of the glutathione-mediated metabolism of glycidamide in mammary epithelial cells. Glycidamide–glutathione conjugates have been identified in serum of rats [87], and mercapturic acid derivates of glycidamide–glutathione conjugates have been characterized in urine of humans [88]; however, few data have been reported on this conjugate in tissues. If glycidamide is conjugated to glutathione for elimination, glutathione levels were expected to be reduced instead of elevated in COMMA-D cells. However, MCF7 breast cancer cells and CaCo-2 colon cancer cells treated with 0.1 µM of glycidamide led to depleted glutathione levels, while treatment with 1 mM glycidamide significantly increased glutathione levels in MCF7 cells and rescued the depleted glutathione levels in CaCo-2 cells [55]. These results suggest different doses of glycidamide have divergent effects on glutathione levels. Acrylamide can also be conjugated to glutathione for removal and excretion [36], indicating glutathione levels could also be depleted after acrylamide treatment [41,42,43,44,45]. Liver, brain, and kidney tissues had depleted glutathione after acrylamide treatment in vivo [41,42,43,44,45]. However, no differences in glutathione or oxidized glutathione levels were observed in acrylamide-treated cells compared to vehicle-treated cells in this study. The half-life of the acrylamide–glutathione conjugate is approximately one hour in serum [87], indicating changes in glutathione levels may not have been captured at the 24 h timepoint for in vitro acrylamide treatment. Additionally, serum acrylamide–glutathione conjugates have a shorter half-life than glycidamide–glutathione conjugates [87]. These studies suggest that the lack of change in glutathione levels in acrylamide-treated cells may be due to more efficient removal of acrylamide than glycidamide in COMMA-D cells.

Acrylamide treatment enhanced oxidative DNA damage in COMMA-D cells, showing that DNA damage can occur through exposure to both glycidamide and acrylamide. The acrylamide-metabolizing enzyme CYP2E1 utilizes NADPH and H^+^ to reduce oxygen to hydrogen peroxide and O_2_^−^ [89], which promotes oxidative stress and mitochondrial dysfunction [90]. The loss of CYP2E1 in COMMA-D cells reversed acrylamide-induced ROS and oxidative DNA damage detected by FLARE assays, demonstrating that CYP2E1 metabolism of acrylamide was a source of DNA damage following acrylamide exposure. CYP2E1 is also induced by alcohol consumption [91], which is another risk factor for breast cancer [3,4]. In fatty liver disease, oxidative damage to the liver is thought to arise in part from CYP2E1-mediated oxidative stress [92], which is elevated with obesity and excess alcohol consumption [59,93,94,95,96]. Further, alcohol consumption under conditions of obesity worsens the severity of fatty liver disease through CYP2E1 activation in rodent models [97,98,99] and in humans [100]. These studies imply that the breast cancer risk factors of obesity and alcohol intake could interact through CYP2E1-associated oxidative stress to promote tumorigenesis. Genetics may also play a role in how CYP2E1 contributes to oxidative stress and DNA damage. Activating polymorphisms such as the c2 allele, which contains a cytosine-to-thymine and a guanine-to-cytosine transversion before the regulatory region of the CYP2E1 gene, have been shown to enhance the severity of fatty liver disease [94]. Further, an insertion polymorphism in the regulator region of the CYP2E1 gene increases the activity of CYP2E1 protein only under conditions of obesity [101], suggesting that both genotype and environment interact to increase risk for diseases associated with CYP2E1 activation. The addition of CYP2E1 substrates like alcohol and acrylamide may further contribute to disease progression. These studies highlight the importance and complexity of investigating the interaction between multiple risk factors to identify genetic and environmental mechanisms that promote breast cancer.

## 5. Conclusions

Overall, long-term exposure to acrylamide at levels found in the Western diet led to increased DNA damage and oxidative stress in mammary epithelial cells under conditions of obesity. Oxidative DNA damage was enhanced through a CYP2E1-mediated mechanism. Chronic dietary exposure to acrylamide may elevate the risk for breast cancer through mutagenesis from glycidamide and oxidative stress, and additional studies are necessary to understand the impact of acrylamide on DNA damage in human breast tissue of patients with obesity. This work highlights the need for prevention strategies to minimize and eliminate acrylamide from the diet as well as identify obesity interventions. Further, CYP2E may be a beneficial target for chemoprevention strategies to reduce risk associated with dietary acrylamide exposure.

## Data Availability

All data generated or analyzed during this study are included in this published article.

## Supplementary Materials

The following supporting information can be downloaded at: https://www.mdpi.com/article/10.3390/toxics12070484/s1, Figure S1: Weight gain, estrus cycle, and liver *Cyp2e1* expression in lean and obese mice treated with acrylamide; Figure S2: Antioxidant levels in mammary epithelial cells are not impacted by diet or acrylamide treatment; Figure S3: Hydrogen peroxide increases DNA damage and oxidative stress in COMMA-D cells; Figure S4: CYP2E1 overexpression enhances oxidative stress in acrylamide-treated COMMA-D cells; Table S1: Primer sequences used for qRT-PCR analyses.

## References

[1] Sung H., Ferlay J., Siegel R.L., Laversanne M., Soerjomataram I., Jemal A., Bray F. (2021). Global Cancer Statistics 2020: GLOBOCAN Estimates of Incidence and Mortality Worldwide for 36 Cancers in 185 Countries. CA Cancer J. Clin..

[2] American Cancer Society Breast Cancer Facts and Figures 2022–2024. https://www.cancer.org/research/cancer-facts-statistics/breast-cancer-facts-figures.html.

[3] Centers for Disease Prevention and Control What Are the Risk Factors for Breast Cancer?. https://www.cdc.gov/cancer/breast/basic_info/risk_factors.htm.

[4] Nindrea R.D., Aryandono T., Lazuardi L. (2017). Breast cancer risk from modifiable and non-modifiable risk factors among women in Southeast Asia: A meta-analysis. Asian Pac. J. Cancer Prev..

[5] Carpenter C.L., Ross R.K., Paganini-Hill A., Bernstein L. (2003). Effect of family history, obesity and exercise on breast cancer risk among postmenopausal women. Int. J. Cancer.

[6] Bandera E.V., Chandran U., Hong C.C., Troester M.A., Bethea T.N., Adams-Campbell L.L., Haiman C.A., Park S.Y., Olshan A.F., Ambrosone C.B. (2015). Obesity, body fat distribution, and risk of breast cancer subtypes in African American women participating in the AMBER Consortium. Breast Cancer Res. Treat..

[7] World Health Organization Obesity and Overweight. https://www.who.int/news-room/fact-sheets/detail/obesity-and-overweight.

[8] Persson L., Carney Almroth B.M., Collins C.D., Cornell S., de Wit C.A., Diamond M.L., Fantke P., Hassellöv M., MacLeod M., Ryberg M.W. (2022). Outside the safe operating space of the planetary boundary for novel entities. Environ. Sci. Technol..

[9] Turkoz F.P., Solak M., Petekkaya I., Keskin O., Kertmen N., Sarici F., Arik Z., Babacan T., Ozisik Y., Altundag K. (2013). The prognostic impact of obesity on molecular subtypes of breast cancer in premenopausal women. J. BUON.

[10] Sahin S., Erdem G.U., Karatas F., Aytekin A., Sever A.R., Ozisik Y., Altundag K. (2017). The association between body mass index and immunohistochemical subtypes in breast cancer. Breast.

[11] Li X., Li J., Hu Q., Zhang X., Chen F. (2024). Association of physical weight statuses defined by body mass index (BMI) with molecular subtypes of premenopausal breast cancer: A systematic review and meta-analysis. Breast Cancer Res. Treat..

[12] Brouckaert O., Van Asten K., Laenen A., Soubry A., Smeets A., Nevelstreen I., Vergote I., Wildiers H., Paridaens R., Van Limbergen E. (2018). Body mass index, age at breast cancer diagnosis, and breast cancer subtype: A cross-sectional study. Breast Cancer Res. Treat..

[13] Neuhouser M.L., Aragaki A.K., Prentice R.L., Manson J.E., Chlebowski R., Carty C.L., Ochs-Balcom H.M., Thomson C.A., Caan B.J., Tinker L.F. (2015). Overweight, obesity, and postmenopausal invasive breast cancer risk: A secondary analysis of the Women’s Health Initiative Randomized Clinical Trials. JAMA Oncol..

[14] Biglia N., Peano E., Sgandurra P., Moggio G., Pecchio S., Maggiorotto F., Sismondi P. (2013). Body mass index (BMI) and breast cancer: Impact on tumor histopathologic features, cancer subtypes and recurrence rate in pre and postmenopausal women. Gynecol. Endocrinol..

[15] Kaviani A., Neishaboury M., Mohammadzadeh N., Ansari-Damavandi M., Jamei K. (2013). Effects of obesity on presentation of breast cancer, lymph node metastasis and patient survival: A retrospective review. Asian Pac. J. Cancer Prev..

[16] Blair C.K., Wiggins C.L., Nibbe A.M., Storlie C.B., Prossnitz E.R., Royce M., Lomo L.C., Hill D.A. (2019). Obesity and survival among a cohort of breast cancer patients is partially mediated by tumor characteristics. npj Breast Cancer.

[17] Chamberlin T., D’Amato J.V., Arendt L.M. (2017). Obesity reversibly depletes the basal cell population and enhances mammary epithelial cell estrogen receptor alpha expression and progenitor activity. Breast Cancer Res..

[18] Shehata M., Teschendorff A., Sharp G., Novcic N., Russell I.A., Avril S., Prater M., Eirew P., Caldas C., Watson C.J. (2012). Phenotypic and functional characterisation of the luminal cell hierarchy of the mammary gland. Breast Cancer Res..

[19] Hein S.M., Haricharan S., Johnston A.N., Toneff M.J., Reddy J.P., Dong J., Bu W., Li Y. (2016). Luminal epithelial cells within the mammary gland can produce basal cells upon oncogenic stress. Oncogene.

[20] Koren S., Reavie L., Couto J.P., De Silva D., Stadler M.B., Roloff T., Britschgi A., Eichlisberger T., Kohler H., Aina O. (2015). PIK3CA(H1047R) induces multipotency and multi-lineage mammary tumours. Nature.

[21] Van Keymeulen A., Lee M.Y., Ousset M., Brohee S., Rorive S., Giraddi R.R., Wuidart A., Bouvencourt G., Dubois C., Salmon I. (2015). Reactivation of multipotency by oncogenic PIK3CA induces breast tumour heterogeneity. Nature.

[22] Arendt L.M., McCready J., Keller P.J., Baker D.D., Naber S.P., Seewaldt V., Kuperwasser C. (2013). Obesity promotes breast cancer by CCL2-mediated macrophage recruitment and angiogenesis. Cancer Res..

[23] Zhao Y.X., Sun Y.L., Ye J.H., Zhang Y., Shi X.B., Wang J.M., Wu H.Y., Zhang W.J., Yao Y.Z. (2020). The relationship between white adipose tissue inflammation and overweight/obesity in Chinese female breast cancer: A retrospective study. Adv. Ther..

[24] Hillers L.E., D’Amato J.V., Chamberlin T., Paderta G., Arendt L.M. (2018). Obesity-activated adipose-derived stromal cells promote breast cancer growth and invasion. Neoplasia.

[25] Gonçalves R.M., Delgobo M., Agnes J.P., das Neves R.N., Falchetti M., Casagrande T., Garcia A.P.V., Vieira T.C., Somensi N., Bruxel M.A. (2021). COX-2 promotes mammary adipose tissue inflammation, local estrogen biosynthesis, and carcinogenesis in high-sugar/fat diet treated mice. Cancer Lett..

[26] International Agency for Research on Cancer (1994). Acrylamide.

[27] Rifai L., Saleh F.A. (2020). A review on acrylamide in food: Occurrence, toxicity, and mitigation strategies. Int. J. Toxicol..

[28] Friedman M., Levin C.E. (2008). Review of methods for the reduction of dietary content and toxicity of acrylamide. J. Agric. Food Chem..

[29] Cordain L., Eaton S.B., Sebastian A., Mann N., Lindeberg S., Watkins B.A., O’Keefe J.H., Brand-Miller J. (2005). Origins and evolution of the Western diet: Health implications for the 21st century. Am. J. Clin. Nutr..

[30] EFSA Panel on Contaminants in the Food Chain (CONTAM) (2015). Scientific Opinion on acrylamide in food. EFSA J..

[31] United States Food and Drug Administration Acrylamide. https://www.fda.gov/food/process-contaminants-food/acrylamide.

[32] Commission Recommendation (EU) 2019/1888 of 7 November 2019 on the Monitoring of the Presence of Acrylamide in Certain Foods. https://op.europa.eu/en/publication-detail/-/publication/a8e890dc-045b-11ea-8c1f-01aa75ed71a1/language-en.

[33] Lee H.W., Pyo S. (2019). Acrylamide induces adipocyte differentiation and obesity in mice. Chem. Biol. Interact..

[34] Kim S.M., Baek J.M., Lim S.M., Kim J.Y., Kim J., Choi I., Cho K.H. (2015). Modified lipoproteins by acrylamide showed more atherogenic properties and exposure of acrylamide induces acute hyperlipidemia and fatty liver changes in zebrafish. Cardiovasc. Toxicol..

[35] Sumner S.C., Fennell T.R., Moore T.A., Chanas B., Gonzalez F., Ghanayem B.I. (1999). Role of cytochrome P450 2E1 in the metabolism of acrylamide and acrylonitrile in mice. Chem. Res. Toxicol..

[36] Fennell T.R., Friedman M.A. (2005). Comparison of acrylamide metabolism in humans and rodents. Adv. Exp. Med. Biol..

[37] Von Tungeln L.S., Churchwell M.I., Doerge D.R., Shaddock J.G., McGarrity L.J., Heflich R.H., Gamboa da Costa G., Marques M.M., Beland F.A. (2009). DNA adduct formation and induction of micronuclei and mutations in B6C3F1/Tk mice treated neonatally with acrylamide or glycidamide. Int. J. Cancer.

[38] Von Tungeln L.S., Doerge D.R., Gamboa da Costa G., Matilde Marques M., Witt W.M., Koturbash I., Pogribny I.P., Beland F.A. (2012). Tumorigenicity of acrylamide and its metabolite glycidamide in the neonatal mouse bioassay. Int. J. Cancer.

[39] Hansen S.H., Olsen A.K., Søderlund E.J., Brunborg G. (2010). In vitro investigations of glycidamide-induced DNA lesions in mouse male germ cells and in mouse and human lymphocytes. Mutat. Res..

[40] Beland F.A., Olson G.R., Mendoza M.C., Marques M.M., Doerge D.R. (2015). Carcinogenicity of glycidamide in B6C3F1 mice and F344/N rats from a two-year drinking water exposure. Food Chem. Toxicol..

[41] Bin-Jumah M.N., Al-Huqail A.A., Abdelnaeim N., Kamel M., Fouda M.M.A., Abulmeaty M.M.A., Saadeldin I.M., Abdel-Daim M.M. (2021). Potential protective effects of Spirulina platensis on liver, kidney, and brain acrylamide toxicity in rats. Environ. Sci. Pollut. Res. Int..

[42] Abdel-Daim M.M., Abo El-Ela F.I., Alshahrani F.K., Bin-Jumah M., Al-Zharani M., Almutairi B., Alyousif M.S., Bungau S., Aleya L., Alkahtani S. (2020). Protective effects of thymoquinone against acrylamide-induced liver, kidney and brain oxidative damage in rats. Environ. Sci. Pollut. Res. Int..

[43] Sengul E., Gelen V., Yildirim S., Tekin S., Dag Y. (2021). The effects of selenium in acrylamide-induced nephrotoxicity in rats: Roles of oxidative stress, inflammation, apoptosis, and DNA damage. Biol. Trace Elem. Res..

[44] Farag O.M., Abd-Elsalam R.M., Ogaly H.A., Ali S.E., El Badawy S.A., Alsherbiny M.A., Li C.G., Ahmed K.A. (2021). Metabolomic profiling and neuroprotective effects of purslane seeds extract against acrylamide toxicity in rat’s brain. Neurochem. Res..

[45] Acaroz U., Ince S., Arslan-Acaroz D., Gurler Z., Kucukkurt I., Demirel H.H., Arslan H.O., Varol N., Zhu K. (2018). The ameliorative effects of boron against acrylamide-induced oxidative stress, inflammatory response, and metabolic changes in rats. Food Chem. Toxicol..

[46] Zhang J.X., Yue W.B., Ren Y.S., Zhang C.X. (2010). Enhanced fat consumption potentiates acrylamide-induced oxidative stress in epididymis and epididymal sperm and effect spermatogenesis in mice. Toxicol. Mech. Methods.

[47] Adani G., Filippini T., Wise L.A., Halldorsson T.I., Blaha L., Vinceti M. (2020). Dietary intake of acrylamide and risk of breast, endometrial, and ovarian cancers: A systematic review and dose-response meta-analysis. Cancer Epidemiol. Biomark. Prev..

[48] Olesen P.T., Olsen A., Frandsen H., Frederiksen K., Overvad K., Tjønneland A. (2008). Acrylamide exposure and incidence of breast cancer among postmenopausal women in the Danish Diet, Cancer and Health Study. Int. J. Cancer.

[49] Hogervorst J.G., Baars B.J., Schouten L.J., Konings E.J., Goldbohm R.A., van den Brandt P.A. (2010). The carcinogenicity of dietary acrylamide intake: A comparative discussion of epidemiological and experimental animal research. Crit. Rev. Toxicol..

[50] Pedersen G.S., Hogervorst J.G., Schouten L.J., Konings E.J., Goldbohm R.A., van den Brandt P.A. (2010). Dietary acrylamide intake and estrogen and progesterone receptor-defined postmenopausal breast cancer risk. Breast Cancer Res. Treat..

[51] Byers S.L., Wiles M.V., Dunn S.L., Taft R.A. (2012). Mouse estrous cycle identification tool and images. PLoS ONE.

[52] Puppel N., Tjaden Z., Fueller F., Marko D. (2005). DNA strand breaking capacity of acrylamide and glycidamide in mammalian cells. Mutat. Res..

[53] Besaratinia A., Pfeifer G.P. (2004). Genotoxicity of acrylamide and glycidamide. J. Natl. Cancer Inst..

[54] Nixon B.J., Katen A.L., Stanger S.J., Schjenken J.E., Nixon B., Roman S.D. (2014). Mouse spermatocytes express CYP2E1 and respond to acrylamide exposure. PLoS ONE.

[55] Clement F.C., Dip R., Naegeli H. (2007). Expression profile of human cells in culture exposed to glycidamide, a reactive metabolite of the heat-induced food carcinogen acrylamide. Toxicology.

[56] Beland F.A., Mellick P.W., Olson G.R., Mendoza M.C., Marques M.M., Doerge D.R. (2013). Carcinogenicity of acrylamide in B6C3F(1) mice and F344/N rats from a 2-year drinking water exposure. Food Chem. Toxicol..

[57] Cinti S., Mitchell G., Barbatelli G., Murano I., Ceresi E., Faloia E., Wang S., Fortier M., Greenberg A.S., Obin M.S. (2005). Adipocyte death defines macrophage localization and function in adipose tissue of obese mice and humans. J. Lipid Res..

[58] Tanos T., Rojo L., Echeverria P., Brisken C. (2012). ER and PR signaling nodes during mammary gland development. Breast Cancer Res..

[59] Emery M.G., Fisher J.M., Chien J.Y., Kharasch E.D., Dellinger E.P., Kowdley K.V., Thummel K.E. (2003). CYP2E1 activity before and after weight loss in morbidly obese subjects with nonalcoholic fatty liver disease. Hepatology.

[60] Poetsch A.R., Boulton S.J., Luscombe N.M. (2018). Genomic landscape of oxidative DNA damage and repair reveals regioselective protection from mutagenesis. Genome Biol..

[61] Tan X., Grollman A.P., Shibutani S. (1999). Comparison of the mutagenic properties of 8-oxo-7,8-dihydro-2′-deoxyadenosine and 8-oxo-7,8-dihydro-2′-deoxyguanosine DNA lesions in mammalian cells. Carcinogenesis.

[62] Norbury C.J., Zhivotovsky B. (2004). DNA damage-induced apoptosis. Oncogene.

[63] Valencia-Olvera A.C., Morán J., Camacho-Carranza R., Prospéro-García O., Espinosa-Aguirre J.J. (2014). CYP2E1 induction leads to oxidative stress and cytotoxicity in glutathione-depleted cerebellar granule neurons. Toxicol. In Vitro.

[64] Wang Y., Chen Q., Wu S., Sun X., Yin R., Ouyang Z., Yin H., Wei Y. (2023). Amelioration of ethanol-induced oxidative stress and alcoholic liver disease by. Acta Pharm. Sin. B.

[65] Grün F., Blumberg B. (2009). Endocrine disrupters as obesogens. Mol. Cell. Endocrinol..

[66] Chu P.L., Lin L.Y., Chen P.C., Su T.C., Lin C.Y. (2017). Negative association between acrylamide exposure and body composition in adults: NHANES, 2003–2004. Nutr. Diabetes.

[67] Zhan Y., Xiao Y., Guan T., Zhang S., Jiang Y. (2020). Relationship between gestational acrylamide exposure and offspring’s growth: A systematic review and meta-analysis of cohort studies. Public Health Nutr..

[68] Huang M., Zhuang P., Jiao J., Wang J., Zhang Y. (2018). Association of acrylamide hemoglobin biomarkers with obesity, abdominal obesity and overweight in general US population: NHANES 2003-2006. Sci. Total Environ..

[69] Doerge D.R., Young J.F., McDaniel L.P., Twaddle N.C., Churchwell M.I. (2005). Toxicokinetics of acrylamide and glycidamide in B6C3F1 mice. Toxicol. Appl. Pharmacol..

[70] Kadry A.M., Friedman M.A., Abdel-Rahman M.S. (1999). Pharmacokinetics of acrylamide after oral administration in male rats. Environ. Toxicol. Pharmacol..

[71] Doerge D.R., Young J.F., McDaniel L.P., Twaddle N.C., Churchwell M.I. (2005). Toxicokinetics of acrylamide and glycidamide in Fischer 344 rats. Toxicol. Appl. Pharmacol..

[72] Rawi S.M., Marie M.-A.S., Fahmy S.R., El-Abied S.A. (2012). Hazardous effects of acrylamide on immature male and female rats. Afr. J. Pharm. Pharmacol..

[73] Quan W., Lin Y., Xue C., Cheng Y., Luo J., Lou A., Zeng M., He Z., Shen Q., Chen J. (2022). Metabolic perturbations and health impact from exposure to a combination of multiple harmful Maillard reaction products on Sprague-Dawley rats. Food Funct..

[74] Ghorbel I., Elwej A., Chaabene M., Boudawara O., Marrakchi R., Jamoussi K., Boudawara T.S., Zeghal N. (2017). Effects of acrylamide graded doses on metallothioneins I and II induction and DNA fragmentation: Bochemical and histomorphological changes in the liver of adult rats. Toxicol. Ind. Health.

[75] Centers for Disease Control and Prevention Heart Disease and Stroke. https://www.cdc.gov/chronicdisease/resources/publications/factsheets/heart-disease-stroke.htm.

[76] Nichols G.A., Hillier T.A., Brown J.B. (2008). Normal fasting plasma glucose and risk of type 2 diabetes diagnosis. Am. J. Med..

[77] Besaratinia A., Pfeifer G.P. (2005). DNA adduction and mutagenic properties of acrylamide. Mutat. Res..

[78] Ghanayem B.I., McDaniel L.P., Churchwell M.I., Twaddle N.C., Snyder R., Fennell T.R., Doerge D.R. (2005). Role of CYP2E1 in the epoxidation of acrylamide to glycidamide and formation of DNA and hemoglobin adducts. Toxicol. Sci..

[79] Manière I., Godard T., Doerge D.R., Churchwell M.I., Guffroy M., Laurentie M., Poul J.M. (2005). DNA damage and DNA adduct formation in rat tissues following oral administration of acrylamide. Mutat. Res..

[80] Martins C., Oliveira N.G., Pingarilho M., Gamboa da Costa G., Martins V., Marques M.M., Beland F.A., Churchwell M.I., Doerge D.R., Rueff J. (2007). Cytogenetic damage induced by acrylamide and glycidamide in mammalian cells: Correlation with specific glycidamide-DNA adducts. Toxicol. Sci..

[81] Atay Z.N., Calgan D., Ozakat E., Varnali T. (2005). Acrylamide and glycidamide adducts of Guanine. J. Mol. Struct..

[82] Patel A.A., Zhang Y., Fullerton J.N., Boelen L., Rongvaux A., Maini A.A., Bigley V., Flavell R.A., Gilroy D.W., Asquith B. (2017). The fate and lifespan of human monocyte subsets in steady state and systemic inflammation. J. Exp. Med..

[83] Hölzl-Armstrong L., Kucab J.E., Moody S., Zwart E.P., Loutkotová L., Duffy V., Luijten M., Gamboa da Costa G., Stratton M.R., Phillips D.H. (2020). Mutagenicity of acrylamide and glycidamide in human TP53 knock-in (Hupki) mouse embryo fibroblasts. Arch. Toxicol..

[84] Zhivagui M., Ng A.W.T., Ardin M., Churchwell M.I., Pandey M., Renard C., Villar S., Cahais V., Robitaille A., Bouaoun L. (2019). Experimental and pan-cancer genome analyses reveal widespread contribution of acrylamide exposure to carcinogenesis in humans. Genome Res..

[85] Marković Filipović J., Miler M., Kojić D., Karan J., Ivelja I., Čukuranović Kokoris J., Matavulj M. (2022). Effect of acrylamide treatment on Cyp2e1 expression and redox status in rat hepatocytes. Int. J. Mol. Sci..

[86] Kannan N., Nguyen L.V., Makarem M., Dong Y., Shih K., Eirew P., Raouf A., Emerman J.T., Eaves C.J. (2014). Glutathione-dependent and -independent oxidative stress-control mechanisms distinguish normal human mammary epithelial cell subsets. Proc. Natl. Acad. Sci. USA.

[87] Luo Y.S., Long T.Y., Chiang S.Y., Wu K.Y. (2021). Characterization of primary glutathione conjugates with acrylamide and glycidamide: Toxicokinetic studies in Sprague Dawley rats treated with acrylamide. Chem. Biol. Interact..

[88] Fennell T.R., Sumner S.C., Snyder R.W., Burgess J., Spicer R., Bridson W.E., Friedman M.A. (2005). Metabolism and hemoglobin adduct formation of acrylamide in humans. Toxicol. Sci..

[89] Gonzalez F.J. (2005). Role of cytochromes P450 in chemical toxicity and oxidative stress: Studies with CYP2E1. Mutat. Res..

[90] Caro A.A., Cederbaum A.I. (2004). Oxidative stress, toxicology, and pharmacology of CYP2E1. Annu. Rev. Pharmacol. Toxicol..

[91] Harjumäki R., Pridgeon C.S., Ingelman-Sundberg M. (2021). CYP2E1 in Alcoholic and Non-Alcoholic Liver Injury. Roles of ROS, Reactive Intermediates and Lipid Overload. Int. J. Mol. Sci..

[92] National Institute of Diabetes and Digestive and Kidney Diseases Symptoms & Causes of NAFLD & NASH. https://www.niddk.nih.gov/health-information/liver-disease/nafld-nash/symptoms-causes.

[93] Orellana M., Rodrigo R., Varela N., Araya J., Poniachik J., Csendes A., Smok G., Videla L.A. (2006). Relationship between in vivo chlorzoxazone hydroxylation, hepatic cytochrome P450 2E1 content and liver injury in obese non-alcoholic fatty liver disease patients. Hepatol. Res..

[94] Varela N.M., Quiñones L.A., Orellana M., Poniachik J., Csendes A., Smok G., Rodrigo R., Cáceres D.D., Videla L.A. (2008). Study of cytochrome P450 2E1 and its allele variants in liver injury of nondiabetic, nonalcoholic steatohepatitis obese women. Biol. Res..

[95] Morgan K., French S.W., Morgan T.R. (2002). Production of a cytochrome P450 2E1 transgenic mouse and initial evaluation of alcoholic liver damage. Hepatology.

[96] Butura A., Nilsson K., Morgan K., Morgan T.R., French S.W., Johansson I., Schuppe-Koistinen I., Ingelman-Sundberg M. (2009). The impact of CYP2E1 on the development of alcoholic liver disease as studied in a transgenic mouse model. J. Hepatol..

[97] Gopal T., Kumar N., Perriotte-Olson C., Casey C.A., Donohue T.M., Harris E.N., Talmon G., Kabanov A.V., Saraswathi V. (2020). Nanoformulated SOD1 ameliorates the combined NASH and alcohol-associated liver disease partly via regulating CYP2E1 expression in adipose tissue and liver. Am. J. Physiol. Gastrointest. Liver Physiol..

[98] Minato T., Tsutsumi M., Tsuchishima M., Hayashi N., Saito T., Matsue Y., Toshikuni N., Arisawa T., George J. (2014). Binge alcohol consumption aggravates oxidative stress and promotes pathogenesis of NASH from obesity-induced simple steatosis. Mol. Med..

[99] Carmiel-Haggai M., Cederbaum A.I., Nieto N. (2003). Binge ethanol exposure increases liver injury in obese rats. Gastroenterology.

[100] de la Maza M.P., Hirsch S., Petermann M., Suazo M., Ugarte G., Bunout D. (2000). Changes in microsomal activity in alcoholism and obesity. Alcohol. Clin. Exp. Res..

[101] McCarver D.G., Byun R., Hines R.N., Hichme M., Wegenek W. (1998). A genetic polymorphism in the regulatory sequences of human CYP2E1: Association with increased chlorzoxazone hydroxylation in the presence of obesity and ethanol intake. Toxicol. Appl. Pharmacol..

