# Supplementary material for: Obesity Induces DNA Damage in Mammary Epithelial Cells Exacerbated by Acrylamide Treatment through CYP2E1-Mediated Oxidative Stress
Source: Toxics. 2024 Jul 2;12(7):484. doi: 10.3390/toxics12070484 (PMC11281187; doi:10.3390/toxics12070484)
Supplement: Supplementary file 1 [file toxics-12-00484-s001.zip › Figure S1.pdf]

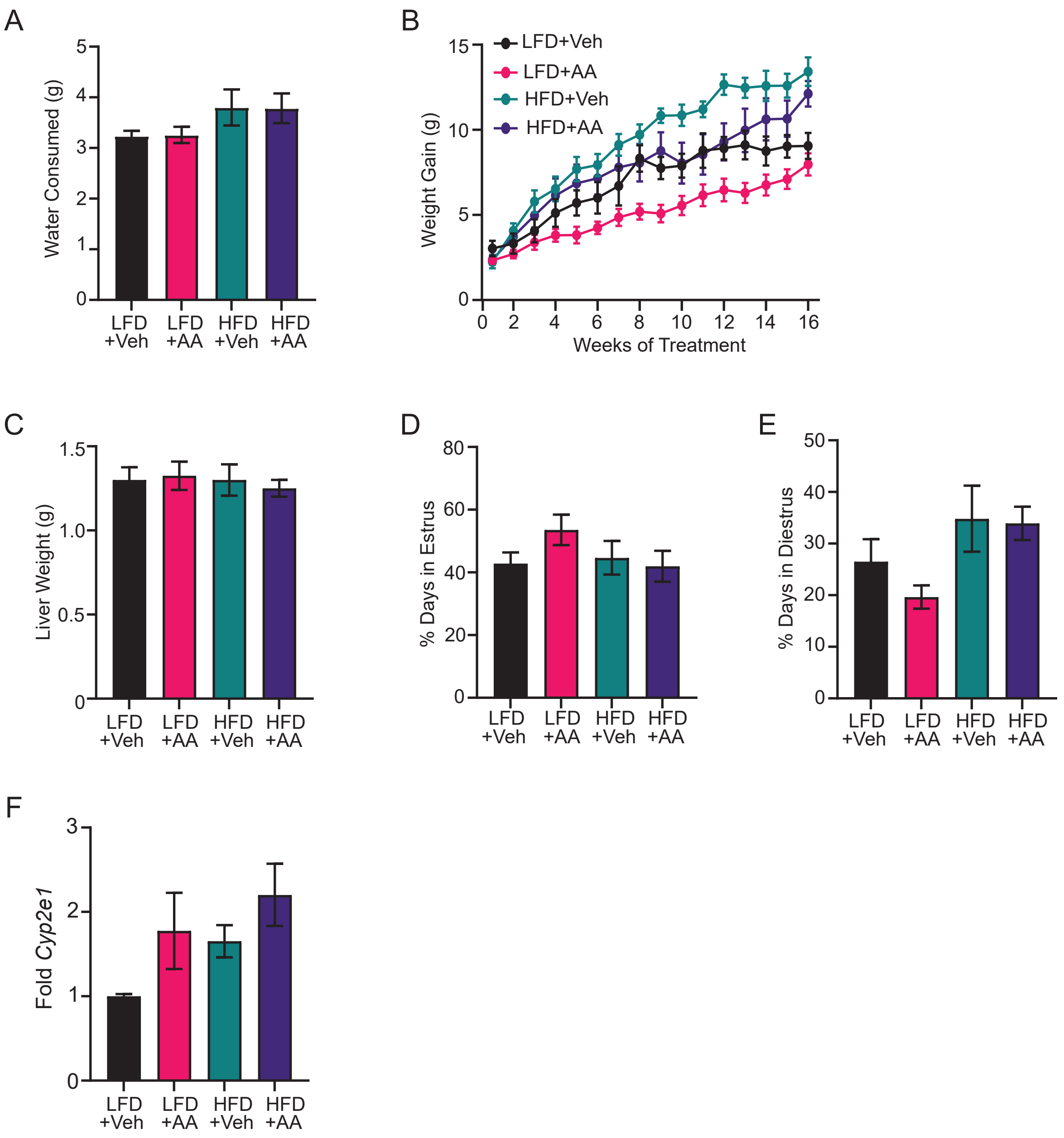

Figure S1. Weight gain, estrus cycle, and liver Cyp2e1 expression in lean and obese mice treated with acrylamide. (A) Water consumption in g of FVB/N female mice fed LFD or HFD with vehicle (veh) or 0.7 mM acrylamide (AA) water (n=5 mice/group). (B) Weight gain of mice fed LFD or HFD over 16 weeks with veh or AA water (n=6-8 mice/group). (C) Liver weights of mice after 16 weeks fed LFD or HFD (n=6-8 mice/group). (D) Quantification of the number of days spent in estrus over two weeks assessed by vaginal cytology (n=6-8 mice/group). (E) Quantification of the number of days spent in diestrus over two weeks assessed by vaginal cytology (n=6-8 mice/group). (F) Relative mRNA expression of Cyp2e1 in the liver (n=3 mice/group). Bars represent mean ± s.e.m.
