# Supplementary material for: Obesity Induces DNA Damage in Mammary Epithelial Cells Exacerbated by Acrylamide Treatment through CYP2E1-Mediated Oxidative Stress
Source: Toxics. 2024 Jul 2;12(7):484. doi: 10.3390/toxics12070484 (PMC11281187; doi:10.3390/toxics12070484)
Supplement: Supplementary file 1 [file toxics-12-00484-s001.zip › Figure S2.pdf]

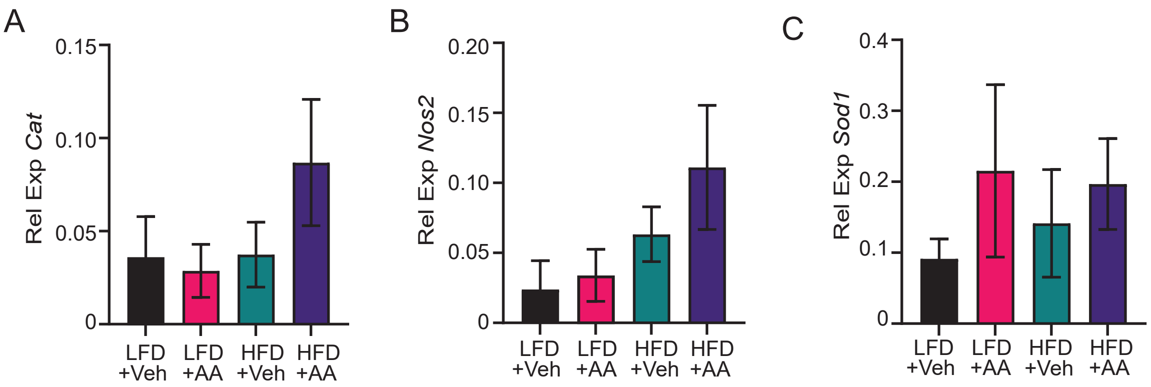

Figure S2. Antioxidant levels in mammary epithelial cells are not impacted by diet or acrylamide treatment. (A) Relative expression of catalase (Cat) mRNA in mammary epithelial cells (n=4-5 mice/group). (B) Relative expression of nitric oxide synthase 2 (Nos2) mRNA in mammary epithelial cells (n=4-5 mice/group). (C) Relative expression of superoxide dismutase 1 (Sod1) mRNA in mammary epithelial cells (n=3 mice/group). Bars represent mean ± s.e.m. Magnification bars = 50 μm.
