# Supplementary material for: Obesity Induces DNA Damage in Mammary Epithelial Cells Exacerbated by Acrylamide Treatment through CYP2E1-Mediated Oxidative Stress
Source: Toxics. 2024 Jul 2;12(7):484. doi: 10.3390/toxics12070484 (PMC11281187; doi:10.3390/toxics12070484)
Supplement: Supplementary file 1 [file toxics-12-00484-s001.zip › Figure S3.pdf]

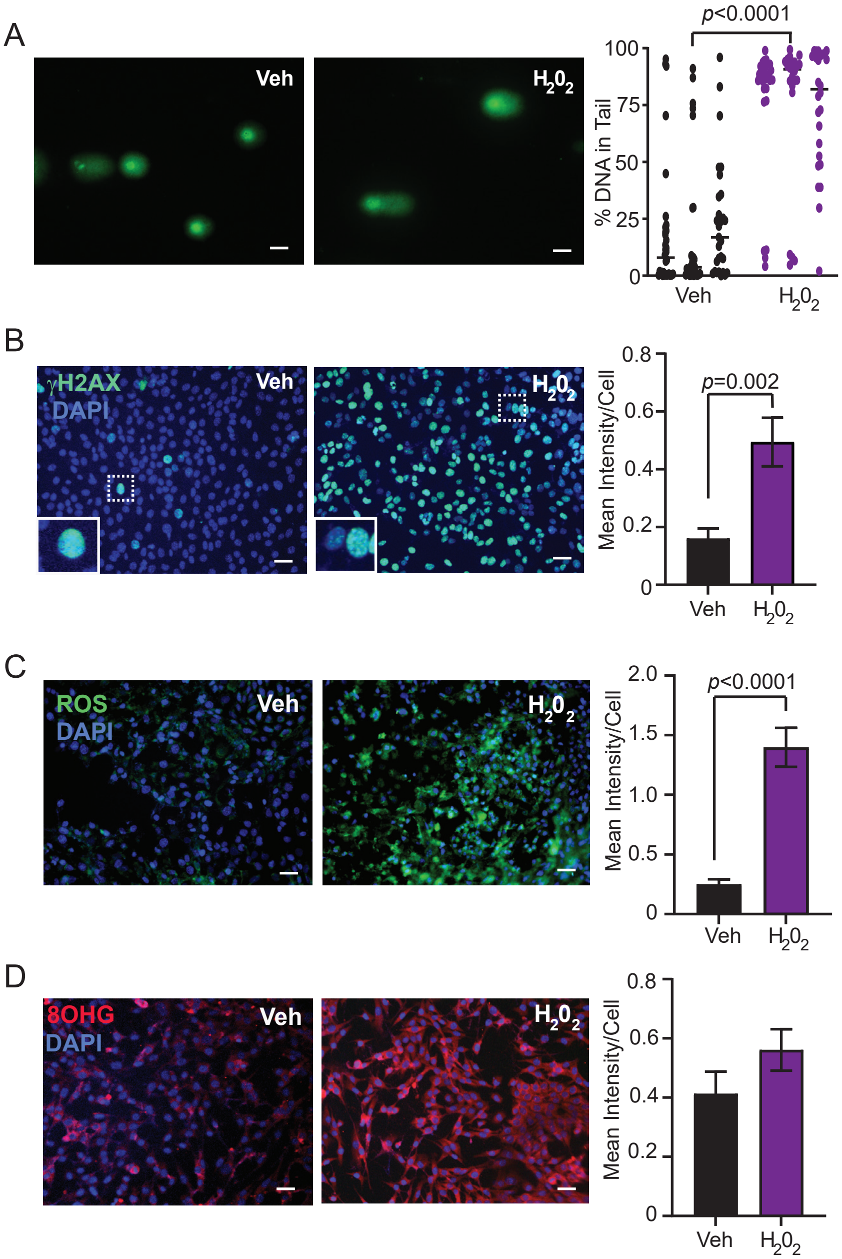

Figure S3. Hydrogen peroxide increases DNA damage and oxidative stress in COMMA-D cells. (A) Representative images of the alkaline comet assay and quantification of the percentage of DNA in the tail of comets in COMMA-D cells treated with 100 mM hydrogen peroxide (H202) for one hour compared to Veh (n=25-35 cells/group/replicate). (B) Representative images and quantification of double strand DNA breaks in H^{2}02-treated cells measured by fluorescent intensity of γH2AX+ cells divided by DAPI+ cells (n=3) images/group/replicate). (C) Representative images and quantification of ROS in Veh and H202 treated cells (n=3 images/well, 2-3 wells/group). (D) Representative images and quantification of oxidized RNA adducts measured by fluorescent intensity of 8-OHG+ cells divided by DAPI+ cells after H202 treatment (n=5 images/group/replicate). Significance between samples were determined using Student's t-test. Bars represent mean ± s.e.m. Magnification bars = 50 μm.
