# Supplementary material for: Obesity Induces DNA Damage in Mammary Epithelial Cells Exacerbated by Acrylamide Treatment through CYP2E1-Mediated Oxidative Stress
Source: Toxics. 2024 Jul 2;12(7):484. doi: 10.3390/toxics12070484 (PMC11281187; doi:10.3390/toxics12070484)
Supplement: Supplementary file 1 [file toxics-12-00484-s001.zip › Figure S4.pdf]

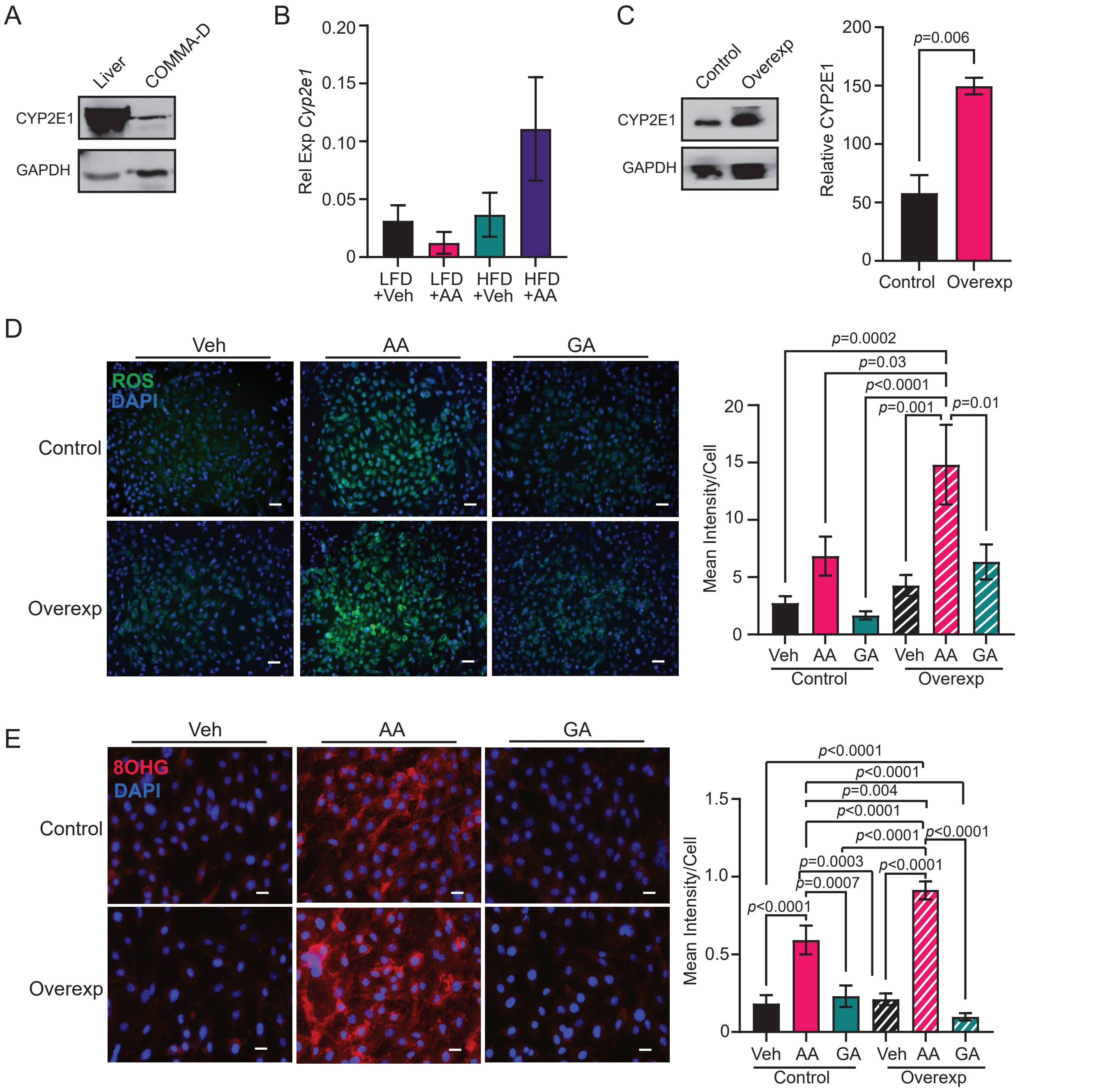

Figure S4. CYP2E1 overexpression enhances oxidative stress in acrylamide-treated COMMA-D cells. (A) Western blot of CYP2E1 and GAPDH protein from mouse liver and COMMA-D cells. (B) Relative expression of Cyp2e1 in mammary epithelial cells from LFD and HFD-fed mice (n=3-5/group). (C) Western blot and quantification of CYP2E1 protein relative to GAPDH in control and CYP2E1 overexpressing COMMA-D cells (n=3 replicates/group). Significance was determined using Student's t-test. (D) Representative images and quantification of ROS in COMMA-D cells overexpressing CYP2E1 compared to control cells treated with Veh, AA, or GA (n=3 images/well, 2 wells/group). (E) Quantification of fluorescent intensity of 8-OHG+ cells divided by total DAPI+ cells in CYP2E1 overexpressing COMMA-D cells and normal CYP2E1 expression (n=5 images/well, 3 wells/group). Bars represent mean ± s.e.m. Magnification bars = 50 μm.
