# Supplementary material for: Obesity Induces DNA Damage in Mammary Epithelial Cells Exacerbated by Acrylamide Treatment through CYP2E1-Mediated Oxidative Stress
Source: Toxics. 2024 Jul 2;12(7):484. doi: 10.3390/toxics12070484 (PMC11281187; doi:10.3390/toxics12070484)
Supplement: Supplementary file 1 [file toxics-12-00484-s001.zip › Table S1.pdf]

Table S1. Primer sequences used for qRT-PCR analyses.

Gene	Forward Sequence	Reverse Sequence
HPRT	TGCTGACCTGCTGGATTACA	TTTATGTCCCCCGTTGACTGA
CYP2E1	CGTTGCCTTGCTTGTCTGGA	AAGAAAGGAATTGGGAAAGGTCC
Catalase (*Cat*)	GGTGTGTTTCTGCGGAACAC	TCAGCCTTCAAACGCCATCT
Nitric Oxide Synthase 2 (*Nos2*)	CCTGTGTGTGTTTCTGCAGC	GCCAGTGATCTACGCGATGA
Superoxide Dismutase 1 (*Sod1*)	GTGGAGAACAGGGTTCTCGG	AGCACCAGGTGGAACAAACA
